# A β-Cyclodextrin-Based Nanoparticle with Very High Transfection Efficiency Unveils siRNA-Activated TLR3 Responses in Human Prostate Cancer Cells

**DOI:** 10.3390/pharmaceutics14112424

**Published:** 2022-11-09

**Authors:** Cristina de la Torre, Pablo Játiva, Inmaculada Posadas, Darío Manzanares, José L. Jiménez Blanco, Carmen Ortiz Mellet, José Manuel García Fernández, Valentín Ceña

**Affiliations:** 1 Unidad Asociada Neurodeath, Facultad de Medicina, Universidad de Castilla-La Mancha, 02006 Albacete, Spain; 2 Centro de Investigación Biomédica En Red (CIBER), Instituto de Salud Carlos III, 28029 Madrid, Spain; 3 Departamento de Química Orgánica, Facultad de Química, Universidad de Sevilla, 41012 Sevilla, Spain; 4 Instituto de Investigaciones Químicas (IIQ), CSIC—Universidad de Sevilla, 41012 Sevilla, Spain

**Keywords:** β-cyclodextrin, glioblastoma, prostate cancer, siRNA, p42-MAPK, Rheb

## Abstract

Synthetic double-stranded small interfering RNAs (siRNAs) mimic interference RNAs (RNAi) and can bind target mRNAs with a high degree of specificity, leading to selective knockdown of the proteins they encode. However, siRNAs are very labile and must be both protected and transported by nanoparticles to be efficiently delivered into cells. In this work, we used a Janus-type polycationic amphiphilic β-cyclodextrin derivative to efficiently transfect siRNAs targeting mRNAs encoding mitogen-activated protein kinase (p42-MAPK) or Ras homolog enriched in brain (Rheb) into different cancer cell lines as well as astrocytes. We took advantage of this high transfection efficiency to simultaneously knock down p42-MAPK and Rheb to boost docetaxel (DTX)-mediated toxicity in two human prostate cancer cell lines (LNCaP and PC3). We found that double knockdown of p42-MAPK and Rheb increased DTX-toxicity in LNCaP but not in PC3 cells. However, we also observed the same effect when scramble siRNA was used, therefore pointing to an off-target effect. Indeed, we found that the siRNA we used in this work induced toll-like receptor 3 activation, leading to β-interferon production and caspase activation. We believe that this mechanism could be very useful as a general strategy to elicit an immune response against prostate cancer cells.

## 1. Introduction

Interference RNA (RNAi) is a powerful physiological mechanism that is active in most cells [1]. Physiologically, RNAi is involved in processing regulatory micro RNAs (miRs), therefore being involved in regulation of cellular metabolism, replication, or malignant transformation [2]. In turn, miRs modulate endogenous gene expression by promoting mRNA degradation if there is perfect complementarity, or, otherwise, they block translation in the more common case that there is limited complementarity [3]. Different synthetic compounds have been used to activate endogenous RNAi mechanisms to interfere with several different pathophysiological mechanisms, many of which have been implicated in an increasing number of diseases. One of these compounds is small interfering RNA (siRNA), which comprises synthetic double-stranded miR mimics that are 20 to 24 base pairs long that, upon binding to the target sequences, induce homologous mRNA degradation [4]. The main advantages of using siRNA technology are its high specificity for mRNA encoding the target protein and the short time required to achieve protein knockdown, which prevents activation of compensatory pathways [5].

Some siRNA-based drugs have already reached clinical settings and have been approved for clinical use by the Food and Drug Administration in the United States. This includes patisiran, where the therapeutic siRNA is encapsulated in a lipid nanoparticle (LNP) carrier to protect it from degradation and to facilitate its uptake by the target cells, and it is intravenously administered to treat hereditary transthyretin-mediated amyloidosis [6], and givosiran, an N-acetylgalactosamine-conjugated RNA interference, which is subcutaneously administered to treat acute hepatic porphyria [7]. LNP delivery systems have been further validated after the worldwide commercialization of the Spikevax^®^ and Comirnaty^®^ vaccines—the first mRNA-based vaccines against the SARS-CoV-2 virus (previously referred to as the Moderna and Pfizer-BioNTech vaccines, respectively)—which were approved by regulatory agencies in 2021 [8,9].

However, these drugs are limited both in terms of their stability and off-target immunogenicity, being difficult to adjust given their multicomponent character and the intrinsic polydisperse nature of some LPN constituents [10,11]. Hence, molecularly defined vectors that are effective in monoformulation formats have emerged as an attractive alternative to LNPs to help researchers realize the full potential of non-viral gene therapy [12,13]. In this sense, cyclic oligosaccharide platforms have proven particularly useful in the production of specifically customized carriers that can then be optimized by precision chemistry strategies that enable configurational and conformational modifications [14,15,16]. Of note, previous work has shown that multi-head/multi-tail Janus-type polycationic amphiphilic β-cyclodextrin (βCD) derivatives can be tailored to undergo coordinated self-assembly alongside genetic material to produce βCD/nucleic acid nanocomplexes (CDplexes) [17,18,19].

Prostate cancer is the second most common cancerous disease type and the fifth leading cause of cancer mortality in men, leading to about 10% of all cancer deaths in men. Advanced prostate cancer accounts for 17% of all diagnosed prostate cancers, with a 5-year survival rate of less than 30% [20]. Until 2015, the cornerstone therapy for treatment of metastatic prostate cancer consisted of androgen deprivation therapy [21]. However, this treatment is not curative and these patients go on to develop metastatic castration-resistant prostate cancer. There has been great interest in the use of taxane-derived antimitotic chemotherapy drugs, such as docetaxel (DTX) combined with androgen inhibitors, after the survival benefit noted with this approach in metastatic hormone-sensitive prostate cancer patients in two large clinical trials, CHAARTED and STAMPEDE [22]. Nevertheless, relapse after these state-of-the-art treatments is still often observed, with tumors becoming highly resistant to further therapy, thereby leading to poor patient prognoses. Therefore, there is an urgent need for new therapeutic approaches to treating this disease.

Several regulatory networks are modified in different tumor types, which gives rise to uncontrolled tumoral cell growth [23]. One of these networks, which is also present in prostate cancer, is the mitogen-activated protein kinase (MAPK) signaling pathway [24]. This pathway involves sequential activation of several proteins, including rat sarcoma virus (Ras)- and rapidly accelerated fibrosarcoma (Raf)-related proteins, which, in turn, lead to activation of MAPKs (especially ERK-1 and ERK-2, with the latter also being known as p42-MAPK) [25]. The mammalian target of the rapamycin (mTOR) signaling pathway is also altered in prostate cancer, which enables proliferation and survival of tumoral cells [26]. Furthermore, guanosine triphosphatase (GTPase) and Ras homolog enriched in brain (Rheb) play important roles in mTOR activation [27]. Consequently, key proteins in these signaling pathways (such as p42-MAPK and Rheb) are appealing targets for interfering with tumoral proliferation and survival by using siRNA interventions [28].

In this current study, we showed that a polycationic amphiphilic βCD derivative, AMC11, is highly biocompatible, strictly monodisperse, and reversibly forms supramolecular CDplexes with siRNA that fully protect the cargo from RNAses-mediated degradation. In addition, here, we report that AMC11 promotes outstanding transfection efficiency in two human prostate cancer cell lines (LNCaP and PC3). We took advantage of the favorable properties of AMC11–siRNA formulations to knock down p42-MAPK and/or Rheb proteins in LNCaP and PC3 cells in order to explore whether reducing the levels of proteins involved in tumoral cell proliferation and survival signaling pathways could boost the anticancer effects of DTX, a drug commonly used in prostate cancer treatment. In addition, we also dissected the siRNA-activated signaling pathways leading to activation of cellular defense mechanisms against foreign RNAs in prostate cancer cells. This will enable future biochemical studies on the mechanisms at play during foreign siRNA internalization and could open new avenues for the development of new therapeutic approaches.

## 2. Materials and Methods

### 2.1. Molecular Vector Synthesis

General methods for AMC11 synthesis are indicated in the Supplementary Materials. AMC11 combines seven tetraethyleneimine branches attached to the primary (narrower) rim of the basket-shaped βCD scaffold through thioureidocysteaminyl connectors and fourteen hexanoyl tails at the opposite secondary face. Its preparation entails selective azidation of the primary positions in commercially available βCD, followed by exhaustive hexanoylation of the secondary hydroxyls with hexanoic anhydride, isothiocyanation, multi-thiourea click-coupling with tetra-N-Boc-protected tetraethyleneimine, and, finally, acid-promoted carbamate hydrolysis. Structural perfection was gauged by 1H and ^13^C NMR spectroscopy, mass spectrometry, and combustion analysis (Figures S1 and S2) [29].

### 2.2. Dynamic Light Scattering (DLS)

The size of the nanoparticles, either alone or coupled to siRNAs, was determined as previously described [30] using a Zetasizer Nano ZS (Malvern Instruments, Malvern, United Kingdom) at 25 °C. The data were analyzed using the multimodal number distribution software included with the instrument. The results are reported as the intensity distribution of the major population by the mean diameter alongside its standard deviation. Ζ-potentials were measured using phase analysis light scattering (M3-PALS) as indicated by the manufacturer. As control, we used either 90 nm monodisperse latex beads (Coulter) for DLS or DTS 50 standard solution (Malvern) for ζ-potential determination.

### 2.3. Transmission Electron Microscopy

Grids (Cu, 200 mesh) were negatively stained by uranyl acetate (1%). After being dried (24 h; room temperature), the grids were placed on CDplex samples (in 20 mm HEPES at pH 7.4). Grids were then dried and imaged using a Philips CM200 electron microscope. Measurements were performed in duplicate at the Centre of Biological Research Services (CIBMS-CSIC) in Madrid (Spain).

### 2.4. Gel Retardation Assay

Direct siRNA/AMC11 interaction was studied following the previously described procedure [31]. Nanoparticle–siRNA complexes were prepared at increasing protonable nitrogen/phosphorous (N/P) ratios, incubated for 30 min at room temperature, and loaded onto a 1.2% agarose gel containing 0.004% (*v*/*v*) Red Safe™ (Intron Biotech, Seoul, South Korea) in TAE buffer (40 mM Tris base, 20 mM glacial acetic acid, and 1 mM EDTA at pH 8.6). Samples were electrophoresed (60 V for 20 min) and photographed under UV-illumination. The sample intensities were determined using ImageJ software [32].

### 2.5. Small Interfering RNA Protection against RNAses

siRNA protection against ribonuclease (RNase)-mediated degradation was studied as previously described [33]. Briefly, N/P–siRNA complexes were prepared at an AMC11–siRNA–N/P ratio of 6.66 by incubating 1 µM AMC11 and 100 nM siRNA for 30 min at room temperature. Complexes, or naked siRNA (100 nM), were incubated in the presence of 0.25% RNase A (Sigma, Barcelona, Spain) for 30 min at 37 °C. The RNase was then inactivated by cooling the samples in an ice-cold water bath for 20 min followed by incubation at 4 °C with heparin (0.5 USP units) to release the siRNA. Next, the samples were processed and the sample bands were quantified as described above.

### 2.6. Cell Culture

LnCaP and PC3 human prostate cancer cell lines, as well as human (U87 MG) and rat (C6) glioblastoma cell lines, were obtained from ATCC (Manassas, VA, USA). Furthermore, GL-261 mouse glioma cells were obtained from the Leibniz Institute DSMZ (Braunschweig, Germany). All the cells were incubated at 37 °C following the provider instructions. Primary astrocytes were isolated from one-day-old mouse pups and cultured in Dulbecco’s Modified Eagle’s Medium (DMEM; Thermofisher; Waltham, MA, USA) supplemented with 10% heat-inactivated fetal bovine serum (FBS), 2 mM L-glutamine, 100 μg/mL streptomycin, and 100 IU/mL penicillin in a saturated humidified atmosphere containing 95% air and 5% CO_2_ maintained at 37 °C. Briefly, the animals were sacrificed by CO_2_ inhalation, decapitated, and the whole brains were extracted. The brain hemispheres were dissected, and the meninges were removed. The hemispheres were chopped with a scalpel blade and digested with trypsin and DNase for 20 min at 37 °C. Fetal bovine serum (10%) was added to stop the enzymatic digestion and the cells were collected by centrifugation and seeded in 24-well plates. The animal experimental study was approved by the Animal Experimentation Ethics Committee at the University of Castilla-La Mancha (UCLM; protocol number PR-2014-10-12) and carried out in accordance with the guidelines from the same UCLM committee and the European Union (directive 2010/63/EU) for the use of laboratory animals.

### 2.7. Toxicity Assay

Late cytotoxicity was determined by measuring the lactate dehydrogenase (LDH) released into the culture medium using the CytoTox96^®^ Non-Radioactive Cytotoxicity Assay kit (Promega, Madrid, Spain) as previously described [34]. Briefly, LDH was measured after 72 h of treatment with either AMC11 (0.1 to 10 µM), DTX (0.1 nM to 1 µM), or CDplexes formed by the different combinations of AMC11 (1 µM) plus either scramble (SCR) siRNA or specific siRNA in the absence or presence of DTX (0.1 nM to 1 µM). The supernatants were collected, the cells lysed using 0.1% (*w*/*v*) Triton X-100 in NaCl (0.9%), and the LDH released into culture media, as well as the LDH cellular content was measured at 490 nm on a 96-well plate reader using a spectrophotometer (Infinite 200, Tecan, Salzburg, Austria). The release of LDH was calculated as the ratio of LDH released/total LDH, with the total LDH being the sum of the LDH content in the culture medium plus the cellular LDH content. The basal toxicity, maximal effect (E_max_), and effective concentration 50 (EC_50_) for DTX in the absence and presence of different siRNAs were calculated by fitting DTX dose–response curves to a log concentration-versus-response model using GraphPad Prism software (La Jolla, CA, USA).

### 2.8. Small Interfering RNA Cellular Uptake

The siRNA cellular uptake was studied as previously described [35]. Briefly, cells were seeded on 20 mm glass coverslips placed in 6-well plates in DMEM medium containing 10% FBS (Gibco, Whatman, MA, USA), antibiotics (penicillin, 100 IU/mL and streptomycin, 100 μg/mL), and L-glutamine (2 mM) and were allowed to attach to the coverslip. CDplexes were prepared by incubating AMC11 (1 µM) for 1 h at room temperature with fluorescently labelled siRNA (FAM-siRNA; 100 nM) in DMEM. Next, 10% FBS, antibiotics, and L-glutamine were added to generate complete medium. The cell culture incubation medium was then replaced with medium containing the CDplexes. Ten minutes before recording the data, Hoechst 33342 (25 µg/mL; Thermofisher, Waltham, MA, USA) was added to the culture medium. At the indicated times, the coverslips were washed 3 times with Krebs–Henseleit (KH) solution (NaCl 140 mM, CaCl_2_ 2.5 mM, MgCl_2_ 1 mM, KCl 5 mM, HEPES 5 mM, and glucose 11 mM at pH 7.4). Fluorescence was recorded using a fluorescence microscope (Nikon Eclipse TE2000-E; Nikon, Tokyo, Japan) and analyzed NIS Elements AR software (Nikon, Tokyo, Japan) [35]. The excitation and emission wavelengths were set at 488 and 520 nm for FAM-siRNA and to 350 and 450 nm for Hoechst 33342, respectively. Intracellular fluorescence intensity quantification was performed using Image J software [32].

### 2.9. Small Interfering RNA Transfection and Western Blot Analysis

Western blot analysis was performed as previously described [34]. Briefly, cells were seeded on 24-well cultured plates as indicated above. CDplexes were prepared by the nanocomplexes formed by AMC11 (1 µM) and the indicated siRNA for 1 h at room temperature with the indicated siRNA concentrations in DMEM. Next, 10% FBS, antibiotics, and L-glutamine were added to generate complete culture medium. Then, the cell culture incubation medium was replaced with medium containing the CDplexes and incubated for 72 h. After that, the cells were then washed 3 times using KH solution, lysed, and the protein content was determined by BCA protein assay (Pierce Biotechnology, Rockford, IL, USA). Protein samples containing 30 µg of protein were loaded onto 12% (p42-MAPK) or 15% (Rheb) SDS-PAGE gels, run at 90 V, and the gels were then transferred to nitrocellulose membranes (Bio-Rad Laboratories, Madrid, Spain). The membranes were blocked in PBS-Tween 20 containing 5% non-fat dry milk and 0.1% BSA protein for 2 h at 4 °C and were then incubated with the corresponding primary antibody overnight at 4 °C. Immunoreactive bands were visualized using an enhanced chemiluminescence system (ECL; Millipore, Madrid, Spain). The immunoblot analysis was performed using the following primary antibodies: polyclonal anti-p42-MAPK (Erk2) antibody (1:500; Cell Signalling Technology, Leiden, The Netherlands), monoclonal anti-Rheb antibody (1:500; R&D Systems, Minneapolis, MN, USA), and monoclonal anti-GAPDH antibody (1:2000; Cell Signalling Technology, Leiden, The Netherlands). Immunoreactive bands were quantified by densitometric analysis using Image J software and the results were expressed as the ratio of the targeted protein density (p42-MAPK or Rheb)/the density of GAPDH, which was used as a loading-control protein. LDH assays and Western blot analysis of the target proteins were performed in the same samples to be sure of the proper protein knockdown in the toxicity experiments.

### 2.10. Isolation of Nuclear and Cytosolic Fractions

PC3 or LnCaP cells were seeded on 6-well plates and, 24 h later, were treated with the vehicle (H_2_O) or AMC11/siRNA nanocomplexes in the absence or presence of (R)-2-(3-chloro-6-fluorobenzo[b]thiophene-2-carboxamido)-3-phenylpropanoic acid, a Toll-Like Receptor 3 (TLR3)-dsRNA complex inhibitor (TI; 10 µM), for 60 min. Afterwards, cells were washed and collected by centrifugation. Cell pellets were resuspended in extraction buffer A (KCl 10 mM, DTT 1 mM, NaF 5 mM, HEPES 10 mM, EDTA 1 mM, EGTA 1 mM, Na_3_VO_4_ 1 mM, Leupeptin 1 mg/mL, Aprotinin 0.1 mg/mL, and PMSF 0.5 mM at pH 8). Nuclei were sedimented by centrifugation at 20,000× *g* for 30 s at 4 °C. Supernatants containing cytosolic extracts were stored at −80 °C. Pellets containing nuclei were resuspended in 50 µL of Buffer B (NaCl 0.4 M, DTT 1 mM, HEPES 20 mM, EDTA 1 mM, EGTA 1 mM, NaF 5 mM, Na_3_VO_4_ 1 mM, leupeptin 1 mg/mL, aprotinin 0.1 mg/mL, and PMSF 0.5 mM at pH 8). Samples were shaken (30 min; 4 °C) and centrifuged at 20,000× *g* for 15 min. The supernatants containing nuclear extracts were stored at −80 °C until used.

### 2.11. Caspase Activity

The activity of caspases 3 and 9 was determined as previously described [36]. The PC3 or LnCaP human prostate cancer cells were treated with the vehicle (H_2_O) or nanocomplexes for different amounts of time. In another set of experiments, cells were treated with the vehicle (H_2_O) or nanocomplexes in the absence or presence of the TLR3 inhibitor TI (10 µM) for 72 h. The cells were then washed twice with cold PBS and lysed in lysis buffer containing HEPES (100 mM at pH 7.4), DTT 5 mM, EGTA 5 mM, Nonidet P-40 (0.04% *v*/*v*), and glycerol 20%. Next, the extracts were centrifuged at 5000× *g* (10 min at 4 °C), the supernatants were collected, and the protein content was determined using the BCA protein assay kit (Thermo Fisher Scientific, Waltham, MA, USA) following the manufacturer’s instructions. Cell extracts (40 μg of protein) were incubated in reaction buffer containing the specific fluorescence substrate at 37 °C for 1 h. For caspase 3 activity, the reaction buffer contained: HEPES (25 mM at pH 7.4), 10% sucrose, 0.1% CHAPS, DTT 10 mM, and the fluorescent substrate Z-Asp-Glu-Val-Asp-7-amino-4-trifluoromethylcoumarin (Z-DEVD-AFC, 50 μM). For caspase 9 activity, the reaction buffer contained: HEPES (50 mM at pH 7.4), NaCl 50 mM, 0.1% CHAPS, 5% glycerol, DTT 10 mM, and the fluorescent substrate Ac-Leu-Glu-His-Asp-7-amino-4-trifluoromethylcoumarin (Ac-LEHD-AFC, 50 μM). Cleavage of the AFC fluorophore was determined using a spectrofluorometer measuring at an excitation wavelength of 400 nm and detecting fluorescence at an emission wavelength of 505 nm. Caspase activity was calculated as the units of fluorescence/mg of protein/h and the results were expressed as the time–fold of caspase activity detected in the vehicle-treated cells.

### 2.12. Interferon β-1a Quantification

PC3 or LnCaP cells were seeded on 6-well plates and, 24 h later, were treated with the vehicle (H_2_O) or AMC11–siRNA nanocomplexes for different lengths of time and in the absence or presence of the TLR3 Inhibitor (R)-2-(3-chloro-6-fluorobenzo[b]thiophene-2-carboxamido)-3-phenylpropanoic acid (TI, 10 µM)f or 72 h. Next, the supernatants were collected and centrifuged at 1500× *g* for 10 min at 4 °C. Aliquots of the supernatants were used to quantify the levels of TNFα and IFNβ by ELISA using commercial kits (ImmunoTools, Friesoythe, Germany) following the manufacturer’s instructions.

### 2.13. Statistical Analysis

Non-parametric variance analysis (Kruskal–Wallis test) followed by Dunn’s test were used to evaluate the statistical differences between the groups, where *p* < 0.05 was considered statistically significant. Statistical analyses were performed using GraphPad Prism software.

## 3. Results

### 3.1. Synthesis, Hydrodynamic Diameter, and ζ-Potential of AMC11

**Figure 1:**
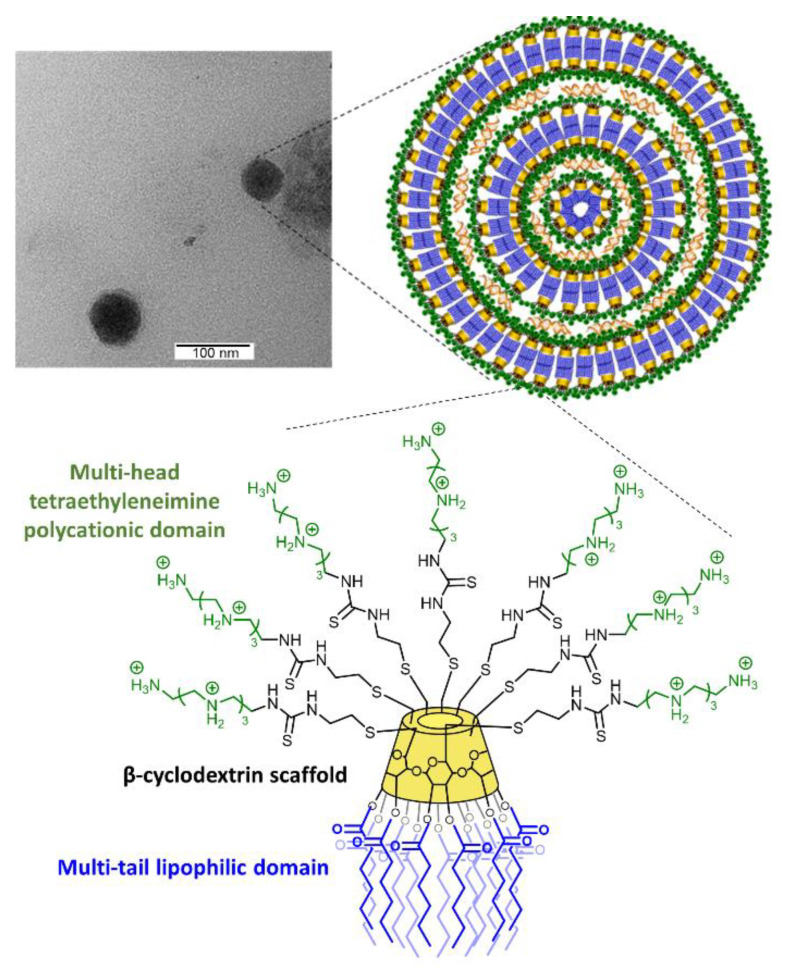
Transmission electron microscopy. TEM image of CDplexes formed by AMC11 (1 µM) and siRNA 100 nM. The cartoon on the right side of the figure shows the most likely organization of the assembled AMC11/siRNA nanocomplexes. The Janus architecture of AMC11 is also shown.

Amphiphilic tetraethyleneimine-β-cyclodextrin molecular vector AMC11 containing 28 protonable nitrogens (Figure 1) was synthesized and characterized as indicated in the Supplementary Materials. In the absence of siRNA and at the concentration used in the biological assays (100 nM), it formed vesicular aggregates with a relatively large size distribution and an average hydrodynamic diameter (measured by DLS) of 170 nm (Figure S3). Upon co-assembly with siRNA, nanocomplexes with an average hydrodynamic diameter of 100 nm were formed (Figure S4). Transmission electron microscopy (TEM) images showed the presence of nanocomplexes with a round shape, which likely consisted of successive layers of AMC11 bound together by siRNA molecules, as indicated in Figure 1. The observed size of 70–80 nm was compatible with the hydrodynamic diameter measured by DLS (100.5 nm). Positive ζ-potential values of 57.6 + 1.39 mV and 25.4 + 0.46 mV were determined for AMC11 aggregates and AMC11/siRNA CDplexes, respectively.

### 3.2. AMC11–Small Interfering RNA Interaction

Cyclodextrin derivative AMC11 is a cationic compound. Accordingly, it was able to bind and completely encapsulate the siRNAs we used at a molar ratio of AMC11–siRNA of 1 µM/100 nM, corresponding to an N/P ratio of 6.66 (Figure S5a). As previously described, binding of cationic species to negatively charged siRNAs is electrostatically driven [31].

Hydrophobic interactions involving the lipid domains facilitates desolvation and reinforces the stability of the nanocomplexes, favoring a lamellar internal order. siRNA binding to AMC11 is reversible because negatively charged macromolecules, such as heparin, can compete with the siRNA cargo and completely promote the release of siRNAs when used at higher concentrations (Figure S5b). Moreover, siRNA bound to AMC11 is protected from degradation by RNases (Figure S5c), thereby further supporting the notion that the corresponding nanocomplexes are suitable for biological experiments.

### 3.3. Cellular Toxicity

**Figure 2:**
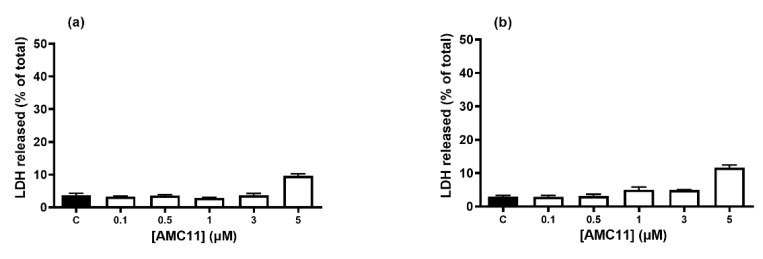
AMC11 toxicity on human prostate cancer cell lines. (**a**) LNCaP and (**b**) PC3 prostate cancer cells were exposed to increasing AMC11 concentrations ranging from 0.1 to 5 µM for 72 h. Then, the percentage of LDH release was determined as an index of cell death. Data represent mean ± SEM of 8 to 12 determinations.

Polycationic macromolecules might have a direct effect on cellular mechanisms and may form the basis of their pharmacological action mechanism [37]. However, when polycationic macromolecules are used as carriers for siRNAs or small drugs, the main effect generally sought is a lack of toxicity. In the case of AMC11, there was no significant toxicity up to 5 µM when used on different cell types, including rat (C6), mouse (GL261), and human (U87) glioblastoma cell lines, primary rat astrocytes (Figure S6), and PC3 and LnCaP human prostate cancer cell lines (Figure 2). Indeed, this finding is consistent with the known biocompatibility of the β-cyclodextrin molecule [37].

### 3.4. Small Interfering RNA Transport into the Cell

**Figure 3:**
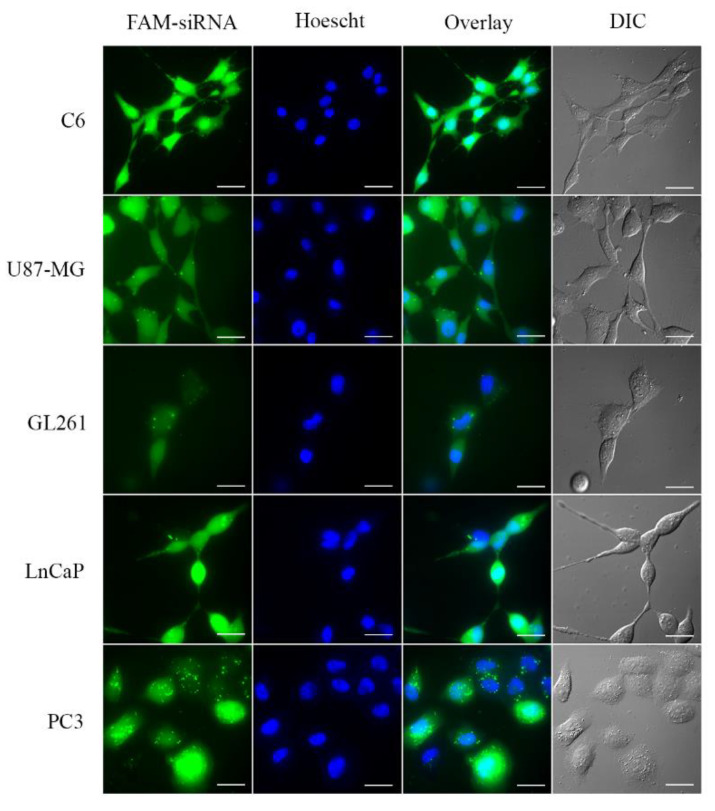
FAM-siRNA cellular uptake in C6, U87, GL261, LNCaP, and PC3 cell lines. AMC11 is capable of efficiently delivering FAM-siRNA into glioblastoma and PC cell lines after 6 or 8 h of incubation. The figure shows representative images of FAM-siRNA, Hoechst 33343 staining the nuclei, overlay of FAM-siRNA, and differential interference contrast microscopy (DIC) image. Scale bar: 25 µm.

In addition to binding and protecting nanoparticles from RNase-mediated degradation, to be an efficient transfection agent, a vector must be able to introduce siRNA inside the cell and facilitate its endosomal escape. As shown in Figure 3, after 6 (C6 cells) or 8 h (U87, GL261, PC3, and LnCaP cells) of treatment with CDplexes comprising AMC11 and FAM-labelled siRNA, all the cell lines studied showed a diffuse but marked increase in fluorescence, although a notable punctate pattern was observed in the case of PC3 cells. This not only indicated that AMC11 was able to transport siRNA to the interior of the cells but also that the AMC11–siRNA complex could escape from the endosomes (punctate pattern) into the cell cytoplasm (diffuse pattern), a step required for protein knockdown. The apparent hydrodynamic diameter of the nanoplexes, as measured by DLS, was about 100 nm, which is highly compatible with endocytosis-mediated uptake into the cell [38].

### 3.5. Transfection Efficiency of AMC11 βCD–Nucleic Acid Nanocomplexes

**Figure 4:**
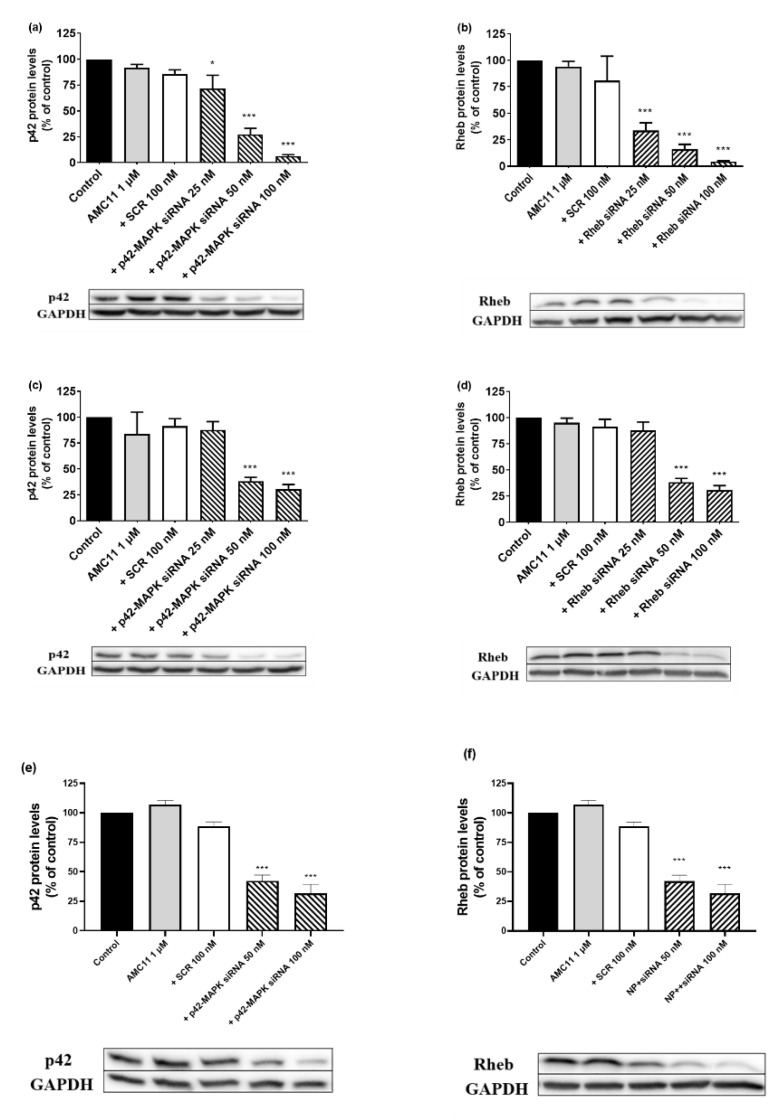
Transfection efficiency of AMC11 in LNCaP, PC3, and astrocytes. (**a**) p42-MAPK protein levels in LNCaP cells, (**b**) Rheb protein levels in LNCaP cells, (**c**) p42-MAPK protein levels in PC3 cells, (**d**) Rheb protein levels in PC3 cells, (**e**) p42-MAPK protein levels in rat astrocytes, and (**f**) Rheb protein levels in rat astrocytes. Cells were exposed for 72 h to the indicated treatments and the specific protein levels determined as indicated in Materials and Methods. Western blot bands show representative experiments for each experimental condition. Data represent mean ± SEM of 5 to 8 experiments: * *p* < 0.05, *** *p* < 0.001 as compared to control values.

Next, we studied the transfection efficiency of AMC11. Incubation of glioblastoma cell lines with AMC11 (1 µM)–siRNA (100 nM) CDplexes caused a significant decrease (ranging from 75 to 90%) in both p42-MAPK (Figure S7) and Rheb (Figure S8) protein levels in C6 rat, GL261 mouse, and human U87 glioblastoma cell lines. In addition, incubation with AMC11-based CDplexes also caused a significant decrease (ranging from 75 to 90%) in p42-MAPK or Rheb protein levels in human prostate cancer LnCaP (Figure 4a,b) or PC3 (Figure 4c,d) cell lines. It is important to note that siRNA delivered by AMC11 also decreased p42-MAPK protein levels by about 65% (Figure 4e) and Rheb (Figure 4f) protein levels by about 75% in rat astrocytes, a primary culture that is usually exceedingly difficult to transfect.

**Figure 5:**
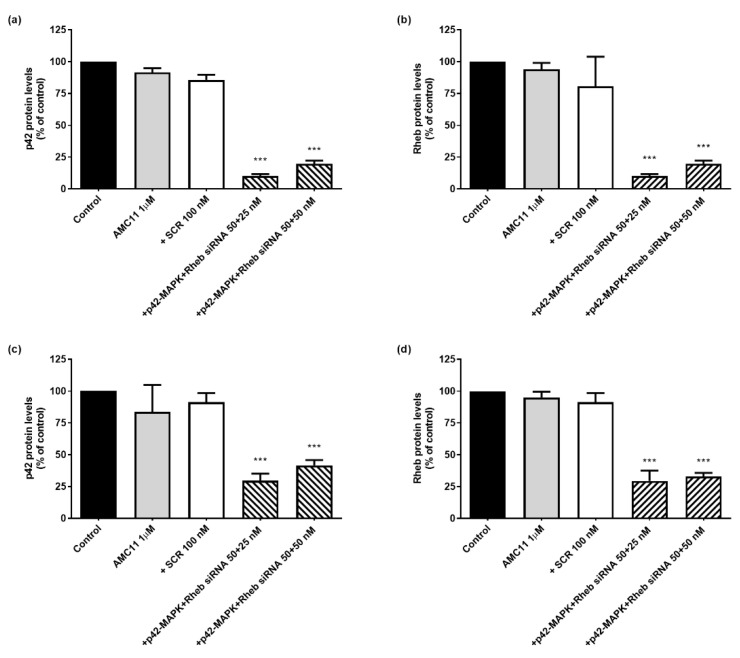
Simultaneous knockdown of p42-MAPK and Rheb in LNCaP and PC3 cell lines. (**a**) p42-MAPK protein levels in LNCaP cells, (**b**) Rheb protein levels in LNCaP cells, (**c**) p42-MAPK protein levels in PC3 cells, and (**d**) Rheb protein levels in PC3 cells. Cells were exposed for 72 h to the indicated treatments and the specific protein levels determined as indicated in Materials and Methods. Data represent mean ± SEM of 4 to 6 experiments *** *p* < 0.001 as compared to control values.

Simultaneous transfection of either LnCaP or PC3 cells with CDplexes formed by AMC11 (1 μM) and specific siRNA (50 nM each) designed to target either p42-MAPK or Rheb mRNA markedly decreased both the target protein levels by the same range as that observed for the individual RNAis (Figure 5). No cross-over effect of siRNAs designed to knock down p42-MAPK on Rheb levels, or vice versa, was observed (data not shown). Control experiments using either AMC11 alone or AMC11–scramble siRNA complexes showed no alteration in p42-MAPK or Rheb protein levels (Figure 5).

### 3.6. Effect of Knocking down Rheb and p42-MAPK on Docetaxel-Induced Cell Death in Prostate Cancer Cell Lines

**Figure 6:**
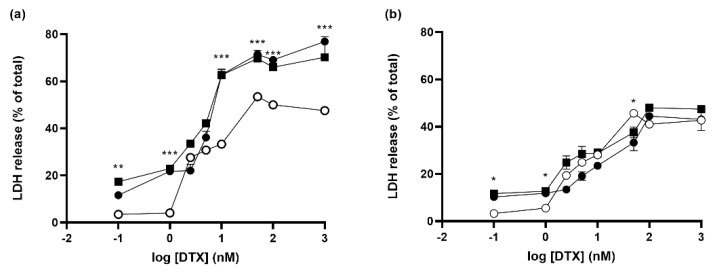
Effect of SCR-siRNA (■; 100 nM) or double p42-MAPK/Rheb knockdown (●) on DTX (○)-induced toxicity in (**a**) LNCaP and (**b**) PC3 cells. Data represent mean ± SEM of 24 to 100 experiments for SCR-siRNA treated or 15 to 240 individual determinations for knockdown experiments. DTX dose–response curves in the absence of siRNA are the same as those shown in Figure S9. If absent, error bars are smaller than the symbol size. * *p* < 0.05, ** *p* < 0.01, *** *p* < 0.001 as compared to DTX in absence of siRNA.

We decided to take advantage of the excellent transfection efficiency of AMC11 to explore whether knocking down two important proteins involved in signaling cancer cell survival and proliferation (Rheb and p42-MAPK) might increase DTX-induced toxicity on human prostate cancer cells. DTX induced dose-dependent toxicity in LnCaP cells, as measured by the release of LDH after 72 h. The quantity of LDH increased from a basal value of 4.2% to a maximum of about 50%, with an EC50 of 7.7 nM (Figure S9; Table 1). When the cells were treated with CDplexes designed to simultaneously knock down Rheb and p42-MAPK, we observed an increase in both background toxicity and maximal cell death, without any significant changes in the DTX EC50 value (Table 1; Figure 6a), suggesting that the toxic action of DTX was indeed boosted. Similar effects were observed when either p42-MAPK or Rheb proteins were knocked down using specific siRNAs (100 nM; Figure S10a; Table 1). However, when SCR siRNA was used as a non-coding control, we observed a higher background toxicity and a slightly lower Emax (Table 1; Figure 6a), pointing to an effect related to the presence of siRNA independent of its coding sequence and unrelated to protein knockdown.

The same experimental approach was used for PC3 cells, which we used as a model for castration-resistant prostate cancer cells [39]. In this case, DTX was slightly less toxic, with a maximal LDH release of about 42% (Figure 6b and Figure S9) and an EC50 of 5.3 nM (Table 2). Dual transfection of PC3 cells with siRNAs targeting p42-MAPK (50 nM) and Rheb (50 nM), in the absence of DTX, significantly increased the background toxicity to about 15% but did not affect the maximal toxicity levels (Figure 6b). However, the EC50 increased to about 14 nM (Table 2), likely reflecting the effect of the increase in basal toxicity caused by siRNA transfection. Treatment of the cells with CDplexes containing only AMC11–SCR siRNA (100 nM) also increased the background LDH levels to a similar extent without influencing the maximal toxic effect (Figure 6b). We also observed an increase in the EC50 (Table 2), and the effects of selectively knocking down p42-MAPK or Rheb using specific siRNAs (100 nM) on the Emax were similar to those obtained after dual p42-MAPK (50 nM) and Rheb siRNA or after SCR siRNA transfection (Figure S10b; Table 2).

### 3.7. Molecular Mechanism of siRNA-Induced Toxicity

**Figure 7:**
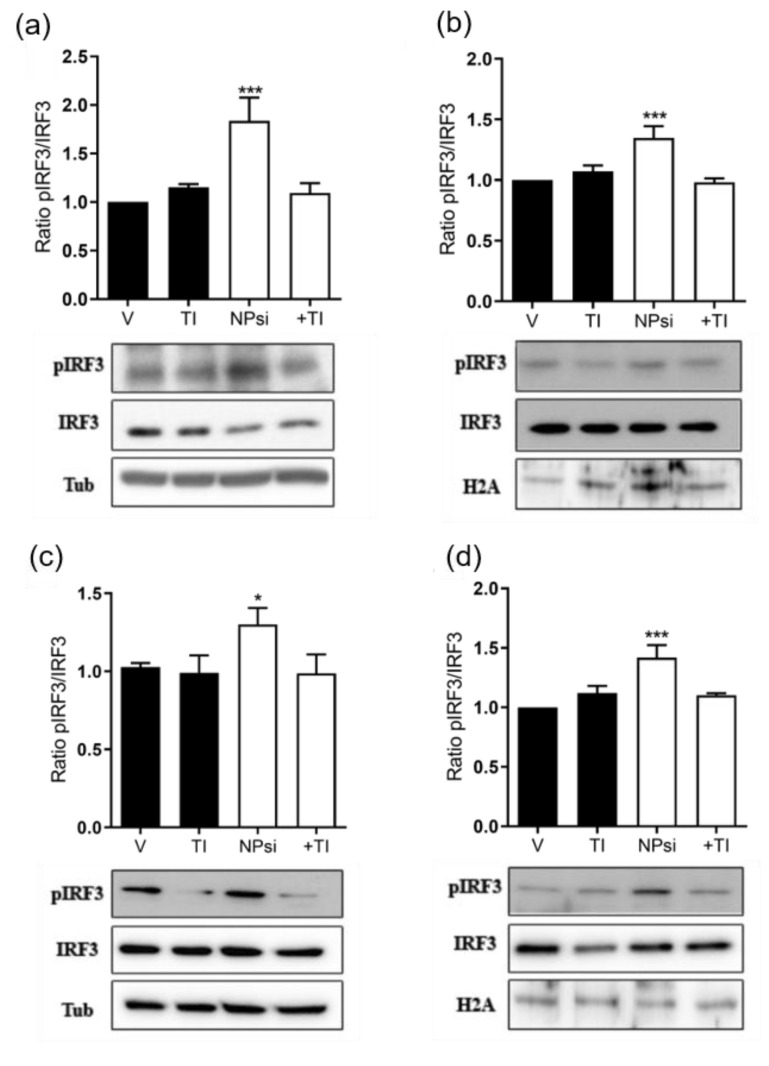
Activation of the TLR3-IRF3 pathway in response to SCR-siRNA administration in LNCaP (**a**,**b**) and PC3 (**c**,**d**) cells. Effect of the TLR3 inhibitor (R)-2-(3-chloro-6-fluorobenzo[b]thiophene-2-carboxamido)-3-phenylpropanoic acid (TI; 10 µM) on phosphorylation of IRF3 (pIRF3) induced by treatment of LnCaP and PC3 cells with nanocomplexes formulated from AMC11 (1 µM) + SCR-siRNA (100 nM) (Npsi) in both cytosolic (**a**,**c**) and nuclear (**b**,**d**) cellular fractions. Data are expressed as mean ± SEM from 6 to 12 determinations from at least 3 independent experiments. * *p* < 0.05, *** *p* < 0.001 compared with vehicle-treated cells (V).

To explore the mechanism involved in siRNA-induced toxicity on the two human prostate cancer cell lines, we studied the signaling pathway involving TLR3-mediated activation of interferon regulatory factor 3 (IRF3), which leads to release of IFNβ and generation of an inflammatory response [40]. IRF3 is activated by phosphorylation and is then translocated into the nucleus for transcription of inflammatory mediators. We found that the pIRF3/IRF3 ratio markedly increased in nuclear and cytosolic fractions of both LNCaP and PC3 cells in response to administration of AMC11–SCR siRNA (Figure 7). However, this increase was completely abolished by the presence of the TLR3 signaling pathway inhibitor (TI; 10 µM).

**Figure 8:**
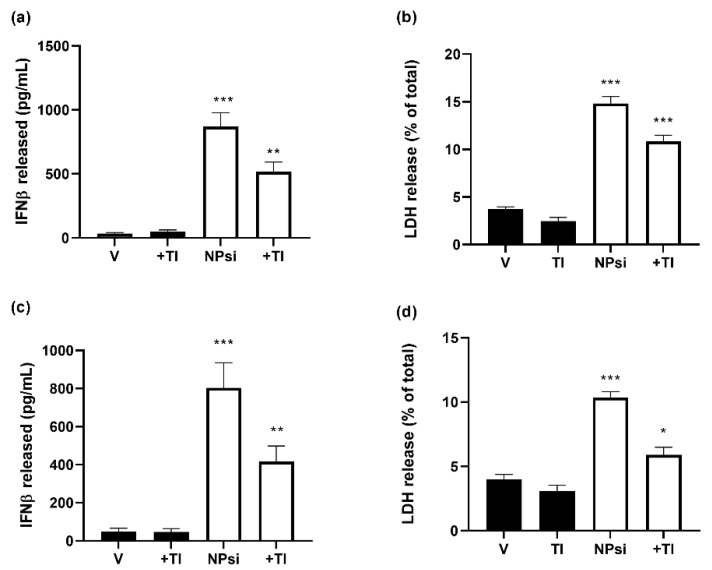
SCR-siRNA-induced production of IFNβ and cell death in LNCaP (**a**,**b**) and PC3 (**c**,**d**). Cells were treated with nanocomplexes formed by AMC11(1 µM) and SCR-siRNA (NPsi, 100 nM) for 72 h, and IFNβ production (**a**,**c**) and cell death (**b**,**d**) were determined in presence and absence of a TLR3 inhibitor (TI, 10 µM). Data are expressed as mean ± SEM from 6 to 12 experiments. * *p* < 0.05; ** *p* < 0.01; *** *p* < 0.001 compared with control-treated cells.

Treatment of both LNCaP and PC3 cells with SCR–siRNA (100 nM) delivered by AMC11 caused a marked time-dependent increase in the release of INFβ (Figure S11). This increase was also partially blocked in the presence of the TLR-3 inhibitor. A similar effect was observed for siRNA-induced LDH release, indicating that, at least in part, siRNA-mediated LNCaP and PC3 cell death was TLR-3-activation-dependent (Figure 8). SCR-siRNA treatment also increased caspase 3 and 9 activities at 24 h, and the increase was completely blocked by the TLR3 inhibitor (TI, 10 µM; Figure S12).

## 4. Discussion

Nanotechnology is a multidisciplinary and innovative science that has been exponentially growing in several fields over the last few decades. More precisely, application of nanotechnologies in medical fields is a promising strategy for approaching personalized medicine and gene therapies [41]. As indicated by the key role lipid nanoparticles play in protecting the mRNA encoding the SARS-CoV-2 antigens used in COVID-19 vaccines [8,9], these applications have already reached clinical settings. Indeed, there are a wide array of applications of nanotechnology in biomedicine, from diagnostics [42,43] to drug and gene delivery [30]. Specifically, the use of nanosystems as drug-vectors improves drug bioavailability, facilitates immune system evasion [44], and provides targeting properties [45]. Furthermore, non-viral carriers represent an excellent alternative to their viral counterparts for delivering genetic materials because they avoid the adverse effects associated with viral vectors [46]. The first limiting step in using gene therapy is finding an effective vector for complexing nucleic acids that will preserve their stability and prevent their degradation until delivery to the target cells. This is especially relevant in the case of RNA molecules (e.g., mRNAs and siRNAs).

The β-cyclodextrin-based molecular vector AMC11 protects siRNA from RNase-mediated degradation, a key property required for effective in vivo transfection to allow in vivo applications of both the AMC11 vector and next-generation AMC11-derived CDplexes to be explored. This protective action is presumably related to a decrease in the solvent-accessible surface area of siRNAs after self-assembly with AMC11, thereby limiting the ability of the active site of RNases to interact with siRNAs, as previously described for other siRNA/molecular vector nanocomplexes [47]. AMC11 also showed no cell toxicity and excellent siRNA transfection capabilities since AMC11/siRNA CDplexes were able to reduce the intracellular levels of either p42-MAPK or Rheb protein to 10 to 25% of the control values in all the cell lines we examined with this degree of protein level reduction, likely producing a lack-of-function for the indicated protein. In addition, AMC11/siRNA CDplexes were also able to decrease the protein levels of both p42-MAPK and Rheb by 65–70% in primary cultures of rat astrocytes. This type of reduction is typically challenging to achieve because all primary cell cultures are difficult to transfect. Moreover, AMC11 was able to simultaneously transfect two different siRNAs into prostate cancer cells to knock down both the p42-MAPK and Rheb protein levels to similar levels to those achieved in single transfection experiments. This confirms that the excellent transfection properties of this molecular vector allow, in this particular case, simultaneous knockdown of key proteins in different signaling pathways.

Interestingly, our hydrodynamic diameter measurements (169.7 nm) indicated that AMC11 aggregates in the absence of siRNA and that the size of the aggregates and CDplexes obtained upon formulation with siRNA (100.5 nm) is compatible with endocytic uptake by cells [38], as shown in Figure 3. This size range clearly indicates that the presumed electrostatic repulsions between the cationic clusters in the vector do not prevent assembly of the individual AMC11 molecules. Indeed, the TEM images showed that co-assembly with siRNA renders spherical supramolecular nanocomplexes. The observed size decrease when going from free AMC11 aggregates to AMC11–siRNA CDplexes concurs with the theory that the positive charges in the vector are partially neutralized by the negatively charged siRNA molecules, thus limiting repulsions between AMC11 branches in a compact multilamellar arrangement.

On the one hand, p42-MAPK (Erk2) is a kinase member of the Ras-Raf-Mek-Erk1/2 signaling pathway involved in cell proliferation, growth, and survival of cancer cells, and it is related to cancer progression and drug-resistance mechanisms in different cancer types, including in prostate cancer [24,48]. Of special note, disruption of this signaling pathway has been linked to better outcomes in the development of anti-cancer therapies [49]. On the other hand, the PI3K-Akt-mTOR-S6K pathway is regulated by Rheb activity [26]. Rheb is a GTP-binding protein with GTPase activity that stimulates the kinase activity of mTOR, leading to activation of a phosphorylation cascade that promotes the protein synthesis required for cell growth and proliferation. This is achieved by activating ribosomal protein S6K [50], which is required for cell proliferation and migration and is also involved in DTX-resistance [51]. Moreover, it has been reported that Rheb expression is increased in prostate cancer cell lines compared to non-malignant cells and that its overexpression promotes cancer progression [51].

In this current work, we took advantage of the excellent siRNA transfection efficiency of AMC11 to simultaneously knock down both p42-MAPK and Rheb proteins. This was completed to prevent these two pathways from compensating each other when one of them was blocked. Thus, we were able to interfere with two of the main proliferation and survival pathways of prostate cancer cells. We hypothesized that dual p42-MAPK and Rheb knockdown would boost the toxic effect of DTX, a taxane drug that inhibits microtubule depolymerization, which is one of the first-line antitumoral drugs used in prostate cancer treatment. Their simultaneous knockdown significantly boosted the maximum toxic effect of DTX in human LnCaP cells, which are considered a good model for the early stages of androgen-dependent prostate cancer [52]. In contrast, no such effect was observed in human PC3 cells, a model for more advanced castration-resistant prostate cancer [53]. Moreover, this increased toxicity occurred without a significant change in the DTX EC50.

The fact that siRNA did not increase the toxic effects of DTX in PC3 cells might be because this cell type is more resistant to taxanes compared to LNCaP cells. Moreover, unlike LNCaP cells, they might have acquired adaptative mechanisms to compensate for the knockdown of p42-MAPK and/or Rheb. However, intensification of DTX-induced LNCaP cell death by siRNA was also present when non-coding scramble siRNA was used, indicating that the effect was related to the presence of the siRNA and not to the target protein knockdown. In addition, we observed a significant increase in basal toxicity (about 15%) in the absence of DTX when siRNA was transfected into both LNCaP and PC3 cells. Taken together, these results suggest that these siRNAs may be producing an off-target effect.

Despite the specific gene silencing selectivity of RNAi mechanisms, the use of siRNA technologies can also produce immunological off-target effects both in vitro and in vivo. Indeed, dsRNAs, such as siRNAs, are recognized by TLRs, mainly TRL3, located in the internal membrane of endosomes [54], although some reports also suggest a possible plasma membrane location [55], which activates multiple transcription factors, including NF-κB, IRF 1/3/7, and c-Jun N-terminal protein kinase, which stimulate the release of chemokines and pro-inflammatory cytokines, leading to activation of type I interferons [11]. Other possible signaling pathways activated by siRNAs include retinoic acid inducible gene 1 (RIG1), which is an RNA helicase that recognizes ssRNA and dsRNA, and, through the adaptor protein IFNβ promoter stimulator 1 (IPS-1), induces activation of IRF3 and NF-κB, which, in turn, results in the release of IFNβ and pro-inflammatory cytokines [56].

Our data indicate that both pathways were probably activated because a TLR3 inhibitor compound was able to completely block phosphorylation of IRF3, preventing its activation, but was only able to partially block either siRNA-induced LNCaP and PC3 cell death or a siRNA-mediated increase in IFNβ, in both cases in the absence of DTX, suggesting that additional signaling mechanisms are activated in response to siRNA transfection. TLR3 receptors are present in prostate cancer cells as well as in other cancer cell types [57], and its activation has been proposed as a mechanism that could generate an immune response against prostate cancer cells [58,59]. Thus, in addition to the specific siRNA-mediated knockdown of proteins involved in the proliferation and survival of tumoral cells, siRNA cargo delivered by AMC11 could also activate the immune system against tumoral cells by means of a non-selective pathway because of the similarity of the siRNA chemical structure with some viral RNAs.

## 5. Conclusions

In summary, the β-cyclodextrin-based molecular vector AMC11 is biocompatible and efficiently protects siRNA from RNase-mediated degradation, making it a suitable candidate for in vivo studies. CDplexes formulated with AMC11 showed excellent siRNA transfection efficiency, which decreased the intracellular levels of the target proteins we studied (p42-MAPK and Rheb) to about 10 to 25% of the control values in five different cell lines, as well as to about 30% in primary astrocytes. Taking all these findings together, these data indicate that AMC11 is an excellent siRNA carrier prototype that can be further elaborated to generate a next generation of molecular vectors incorporating additional functionalities. We took advantage of this excellent transfection profile to knock down p42-MAPK and Rheb to boost DTX-induced cell death in androgen-dependent LNCaP prostate cancer cells. However, we found that this effect was produced by off-target siRNA activation of TLR3-mediated IFNβ production, as well as caspase activation. Thus, we believe that this mechanism might be very useful in future research as a general strategy to elicit an immune response against prostate cancer cells.

## Figures and Tables

**Table 1:** DTX-induced toxicity and EC50 values. LNCaP prostate cancer cells were exposed to DTX (0.1 nM to 1 µM) alone or in the presence of different siRNA treatments for 72 h and toxicity determined. Individual DTX dose–response curves were fitted to a log concentration versus response model. Data represent mean ± SEM of 5 to 12 experiments.

Treatment	Basal (% LDH Released)	E_max_ (% LDH Released)	EC_50_ (nM)
DTX	4.2 ± 0.1	50.6 ± 1.1	7.7 ± 1.3
DTX + SCR-siRNA (100 nM)	24.9 ± 3.2	68.1 ± 2.4	5.6 ± 0.6
DTX + p42-siRNA (100 nM)	14.3 ± 2.7	78.9 ± 2.2	6.1 ± 1.1
DTX + Rheb-siRNA (100 nM)	15.5 ± 3.7	71.8 ± 2.3	5.9 ± 0.7
DTX + p42-siRNA (50 nM) + Rheb-siRNA (50 nM)	18.3 ± 4.2	73.8 ± 2.1	5.8 ± 0.7

**Table 2:** DTX-induced toxicity and EC50 values. PC3 prostate cancer cells were exposed to DTX (0.1 nM to 1 µM) alone or in the presence of different siRNA treatments for 72 h and toxicity determined. Individual DTX dose–response curves were fitted to a log concentration versus response model. Data represent mean ± SEM of 4 to 7 experiments.

Treatment	Basal (% LDH Released)	E_max_ (% LDH Released)	EC_50_ (nM)
DTX	3.7 ± 0.7	42.7 ± 1.3	5.3 ± 0.6
DTX + SCR-siRNA (100 nM)	14.3 ± 1.7	47.1 ± 1.6	22.1 ± 4.3
DTX + p42-siRNA (100 nM)	12.6 ± 3.2	42.4 ± 3.8	9.1 ± 4.6
DTX + Rheb-siRNA (100 nM)	12.1 ± 2.3	41.2 ± 1.9	8.9 ± 2.7
DTX + p42-siRNA (50 nM) + Rheb-siRNA (50 nM)	15.2 ± 1.9	44.5 ± 3.5	14.1 ± 6.4

## Data Availability

The manuscript data will be available from the authors upon reasonable request.

## Supplementary Materials

The following supporting information can be downloaded at: https://www.mdpi.com/article/10.3390/pharmaceutics14112424/s1, General Methods; Figure S1. ^1^H and ^13^C NMR spectra of AMC8; Figure S2. ^1^H and ^13^C NMR spectra of AMC11; Figure S3. Size distribution of AMC11; Figure S4. Size distribution of the nanoplex formed by AMC11 (1 µM) and SCR-siRNA (100 nM); Figure S5. AMC11 interaction with siRNA; Figure S6. AMC11 toxicity on glioblastoma cell lines and astrocytes; Figure S7. AMC11–siRNA knockdown of p42-MAPK protein levels in glioblastoma cell lines; Figure S8. AMC11–siRNA knockdown of Rheb protein levels in glioblastoma cell lines; Figure S9. Dose–response curve of DTX-induced toxicity in LNCaP and PC3 cells; Figure S10. Effect of p42 or Rheb knockdown on DTX-induced toxicity in LNCaP and PC3 cells; Figure S11. Time-course SCR-siRNA-induced production of IFNβ in LNCaP and PC3 cells; Figure S12. Scramble-siRNA-induced activation of caspases in LNCaP and PC3 cell lines; original Western blot gels.
