# Supplementary material for: A β-Cyclodextrin-Based Nanoparticle with Very High Transfection Efficiency Unveils siRNA-Activated TLR3 Responses in Human Prostate Cancer Cells
Source: Pharmaceutics. 2022 Nov 9;14(11):2424. doi: 10.3390/pharmaceutics14112424 (PMC9692777; doi:10.3390/pharmaceutics14112424)

SUPPLEMENTARY INFORMATION

General Methods……………………………………………………………………………… 2

Figure S1. ^{1}H and ^{13}C NMR spectra of AMC8……………………………………………… 5

Figure S2. ^{1}H and ^{13}C NMR spectra of AMC8……………………………………………… 6

Figure S3. Size distribution of AMC11……………………………………………………. 7

Figure S4. Size distribution of the nanoplex formed by AMC11 (1 µM) and SCR-siRNA (100 nM)……………………………………………………………….… 8

Figure S5. AMC11 interaction with siRNA………………………………………………. 9

Figure S6. AMC11 toxicity on glioblastoma cell lines and astrocytes………………………. 10

Figure S7. AMC11-siRNA knock down of p42-MAPK protein levels in glioblastoma cell lines…………………………………………………………………… 11

Figure S8. AMC11-siRNA knock down of Rheb protein levels in glioblastoma cell lines…………………………………………………………..……. 12

Figure S9. Dose-response curve of DTX-induced toxicity in in LNCaP and PC3 cells ….. 13

Figure S10. Effect of p42 or Rheb knock down on DTX-induced toxicity in LNCaP and PC3 cells ………………………………………………………………. 14

Figure S11. Time-course SCR-siRNA-induced production of IFNβ in LNCaP and PC3 cells……………………………………………………………..……… 15

Figure S12. Scramble-siRNA-induced activation of caspases in LNCaP and PC3 cell lines………………………………………………………………………… 16

Original western blot gels………………………………………………………………. 17

Figure 5………………………………………………………………………….. 17

Figure 7………………………………………………………………………….. 21

General methods

Optical rotations were measured at 20 ± 2 °C in 1-dm tubes on a Jasco P-2000 polarimeter. Ultraviolet-visible (UV) spectra were recorded in 1-cm tubes on a Jasco V-630 spectrophotometer. Infrared (IR) spectra were recorded on a Jasco ATR MIRacle™ spectrophotometer. ^{1}H (and ^{13}C NMR) spectra were recorded at 500 (125.7) MHz with Bruker 500 DRX. 1D TOCSY, 2D COSY, HMQC and HSQC experiments were used to assist on NMR assignments. Thin-layer chromatography (TLC) was carried out on aluminium sheets coated with Kieselgel 60 F254 (E. Merck), with visualization by UV light and by charring with 10% H_2SO_4. With preparative purposes, column chromatography was carried out on Silica Gel 60 (E. Merck, 230-400 mesh). Electrospray mass spectra were obtained for samples dissolved in MeOH or H2O-MeOH mixtures at low μm concentrations. Elemental analyses were carried out at the Instituto de Investigaciones Químicas (Sevilla, Spain) using an elemental analyser *Leco CHNS-932* o *Leco TruSpec CHN.* Heptakis [6-(2-isothiocyanatoethylthio)-2,3-di-*O*-hexanoyl]-cyclomaltoheptaose (**PBO235**) and N^1,N^2,N^3,N^4-tetra(*tert*-butoxycarbonyl)-tetraethylenepentamine (**AMC1**) were combined to obtain AMC8 or AMC11.

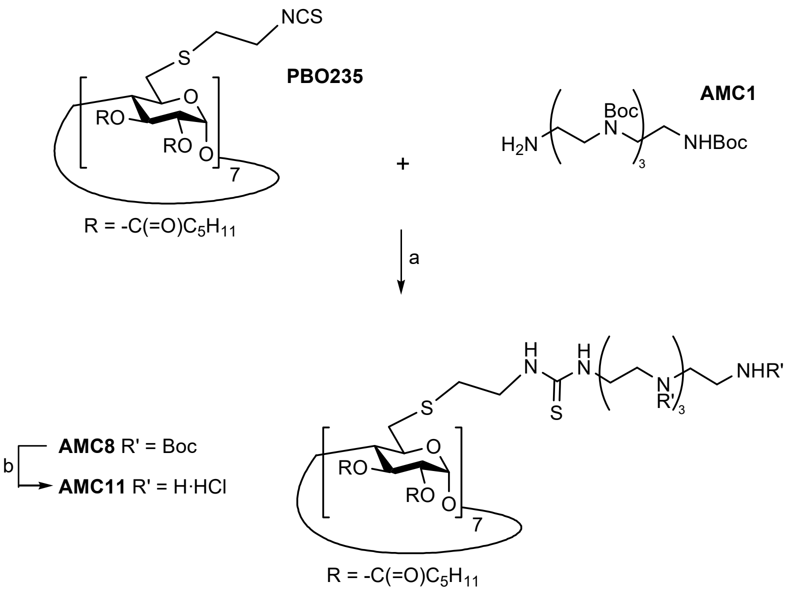

Scheme S1. Synthesis of amphiphilic CD **AMC11**: a) Et_3N, DMF, rt, 2h, 74%; b) 1:1 TFA-DCM, rt, 2h, quantitative.

Heptakis[6-deoxy-2,3-di-*O*-hexanoyl-6[-2[-*N'*-[triethylenetetra[(*tert*-butoxycarbonyl)-amino]ethylene]thioureido]ethyltio]]cyclomaltoheptaose (AMC8). To a solution of **PBO235** (100 mg, 0.031 mmol) and **AMC1** (138 mg, 0.235 mmol) in dry DMF (5 mL), Et_3N (0.030 mL, 0.217 mmol) was added and the reaction mixture was stirred at rt for 2 hours. The solvent was removed under vacuum and the residue purified by column chromatography (25:1 → 20:1 DCM-MeOH) to give **AMC8** as an amorphous solid. Yield: 168 mg (74%); R_f = 0.45 (9:1 CH_2Cl_2:MeOH); [□]$_D$ = +40.7 (*c* 0.97, DCM); UV (DCM): 249 nm (□$_{mM}$ 109.5); IR: □$_{max}$ = 3342, 2971, 2931, 1752, 1697, 1366, 1247, 1164 cm^{-1}; ^1H NMR (500 MHz, CD_3OD, 333 K): δ = 5.35 (t, 7 H, $J_{2,3} = J_{3,4}$ = 8.0 Hz, H-3), 5.18 (d, 7H, $J_{1,2}$= 3.5 Hz, H-1), 4.85 (dd, 7 H, H-2), 4.20 (m, 7 H, H-5), 3.95 (t, 7 H, H-4), 3.77, 3.68 (2 bt, 28 H, CH_2NHCS), 3.46-3.32 (m, 70 H, CH_2), 3.28 (bd, 7 H, $J_{6a,6b}$= 12.0 Hz, H-6a), 3.22 (t, 14 H, $^3J_{H,H}$ = 6.0 Hz, CH_2NHBoc), 3.19 (dd, 7 H, $J_{5,6b}$ = 5.2 Hz, H-6b), 2.94 (m, 14H, CH_2S), 2.46 (m, 14 H, H-2a$_{Hex}$), 2.31 (m, 14 H, H-2b$_{Hex}$), 1.66 (m, 28 H, H-3$_{Hex}$), 1.51, 1.50, 1.46 (s, 63 H, CMe_3), 1.41 (m, 28 H, H-4$_{Hex}$), 1.37-1.31 (m, 28 H, H-5$_{Hex}$), 0.95 (m, 42 H, H-6$_{Hex}$); ^{13}C NMR (125.7 MHz, CD_3OD, 323 K): δ = 183.0 (CS), 173.2, 172.0 (C-1$_{Hex}$), 156.8, 155.9, 155.7 (CO carbamate), 96.8 (C-1), 80.0 (*C*Me_3), 78.6 (C-4), 71.9 (C-5), 70.6 (C-3), 70.3 (C-2), 45.2 (CH_2), 43.8, 42.4 (CH_2NHCS), 38.6 (*C*H_2NHBoc), 33.9 (C-6), 33.8, 33.7 (C-2$_{Hex}$), 32.8 (CH_2S), 31.2, 31.1 (C-4$_{Hex}$), 27.7 , 27.6, 27.5 (C*Me*$_3$), 24.2 (C-3$_{Hex}$), 22.1 (C-5$_{Hex}$), 13.1, 12.9 (C-6$_{Hex}$); ESIMS: *m/z* = 3694.3 [M + 2Na]$^{2+}$. Anal. Calcd for $C_{343}H_{616}N_{42}O_{98}S_{14}$ (7345.56): calcd. C, 56.08; H, 8.45; N, 8.09; found: C, 56.17; H, 8.35; N, 7.84.

Heptakis[6-deoxy-2,3-di-*O*-hexanoyl-6[-2[-*N'*-[triethylenetetramino]ethylene]-thioureido]ethyltio]]cyclomaltoheptaose hydrochloride (AMC11) Compound **AMC8** (138 mg, 0.019 mmol) was treated with TFA-CH_2Cl_2 (1:1, 4 mL) at room temperature for 2 h. Then solvent was evaporated and acid traces removed by co-evaporation with water, and the residue was solved in 10:1 H_2O-HCl 0.1 M and freeze-dried to yield quantitatively **AMC11**. Yield: 104 m; R_f = 0.10 (5:3:5 CH_3CN-H_2O-NH_4OH); $[□]_D$ = +34.3 (*c* 1.05, MeOH); UV (MeOH): 244 nm ($□_{mM}$ 26.42); ^1H NMR (500 MHz, 5:1 CD_3OD-D_2O, 333 K): δ = 5.32 (t, 7 H, $J_{2,3} = J_{3,4}$ = 8.5 Hz, H-3), 5.17 (d, 7H, $J_{1,2}$ = 3.5 Hz, H-1), 4.85 (dd, 7 H, H-2), 4.16 (bs, 7 H, H-5), 3.95 (t, 14 H, $^3J_{H,H}$= 6 Hz, CH_2NHCS), 3.92 (t, 7 H, H-4), 3.74 (bs, 14 H, CH_2NHCS), 3.49-3.32 (m, 84 H, CH_2), 3.25 (bd, 7 H, $J_{6a,6b}$ = 13.0 Hz, H-6a), 3.15 (dd, 7 H, $J_{5,6b}$ = 5.5 Hz, H-6b), 2.92 (m, 14H, CH_2S), 2.45 (m, 14 H, H-$2a_{Hex}$), 2.31 (m, 14 H, H-$2b_{Hex}$), 1.62 (m, 28 H, H-3_{Hex}), 1.41 (m, 28 H, H-4_{Hex}), 1.36 (m, 28 H, H-5_{Hex}), 0.93 (m, 42 H, H-6_{Hex}); ^{13}C NMR (125.7 MHz, 5:1 CD_3OD-D_2O, 333 K): δ = 183.0 (CS), 173.9, 172.3 (C-1_{Hex}), 96.9 (C-1), 78.8 (C-4), 71.9 (C-5), 70.7 (C-3), 70.3 (C-2), 45.7, 45.3, 44.8 (CH_2), 44.2 (CH_2NHCS), 43.9 (CH_2), 40.2 (CH_2NHCS), 34.0(C-6), 33.8, 33.7 (C-2_{Hex}), 32.6 (CH_2S), 31.1, 31.0 (C-4_{Hex}), 24.2, 24.1 (C-3_{Hex}), 22.0 (C-5_{Hex}), 13.2, 13.0 (C-6_{Hex}); ESIMS: *m/z* = 1515.1 $[M + Na + 2 H]^{3+}$. Anal. Calcd for $C_{198}H_{410}N_{42}O_{42}S_{14}Cl_{28}\cdot 24 H_2O$ (6057.71): calcd. C, 41.44; H, 7.62; N, 9.71; S, 7.41; found: C, 41.30; H, 7.60; N, 10.11; S, 7.71.

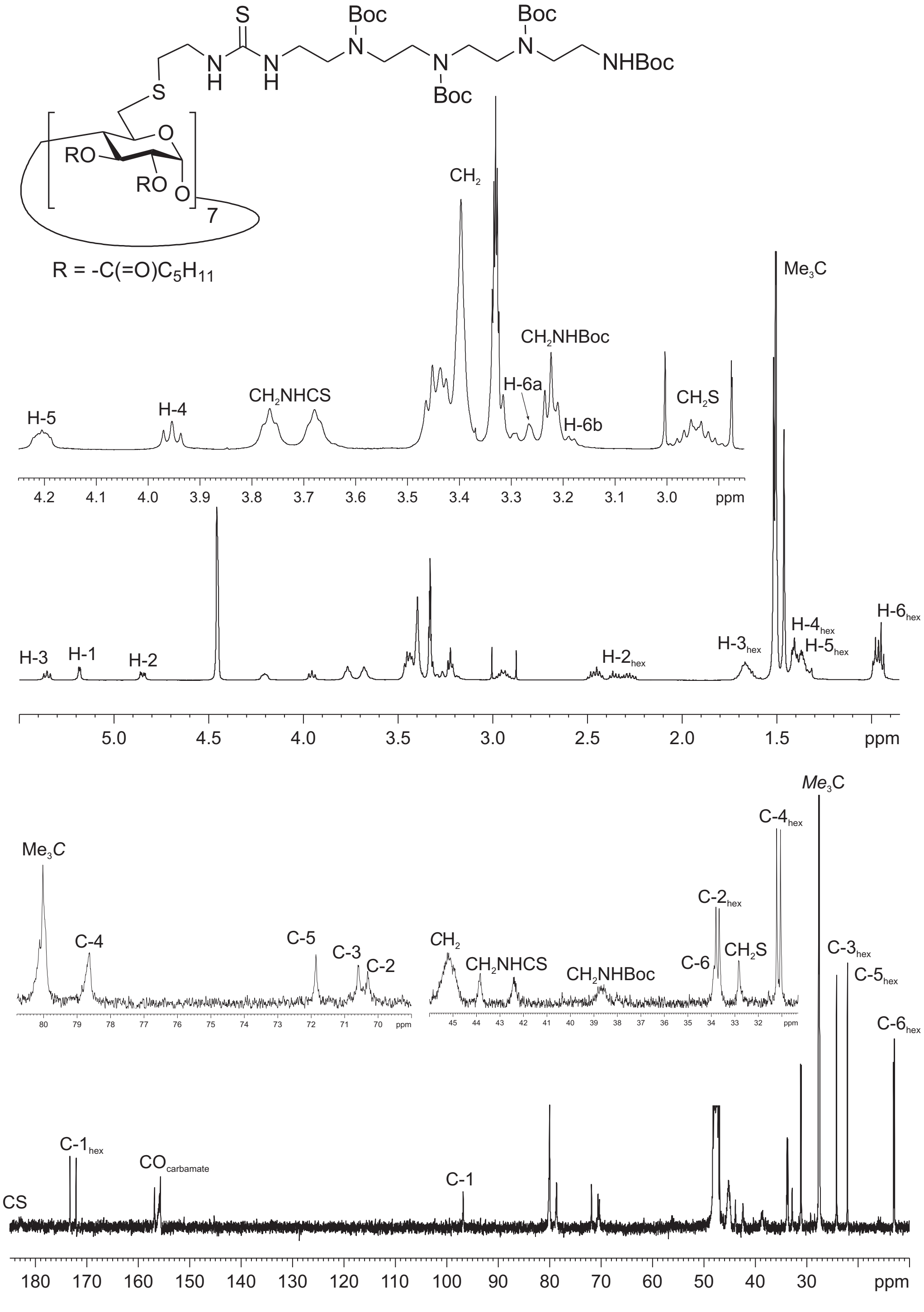

Figure S1. ^{1}H and ^{13}C NMR spectra (500 MHz, 125.7 MHz, CD_3OD, 333 K and 323 K, respectively) of AMC8.

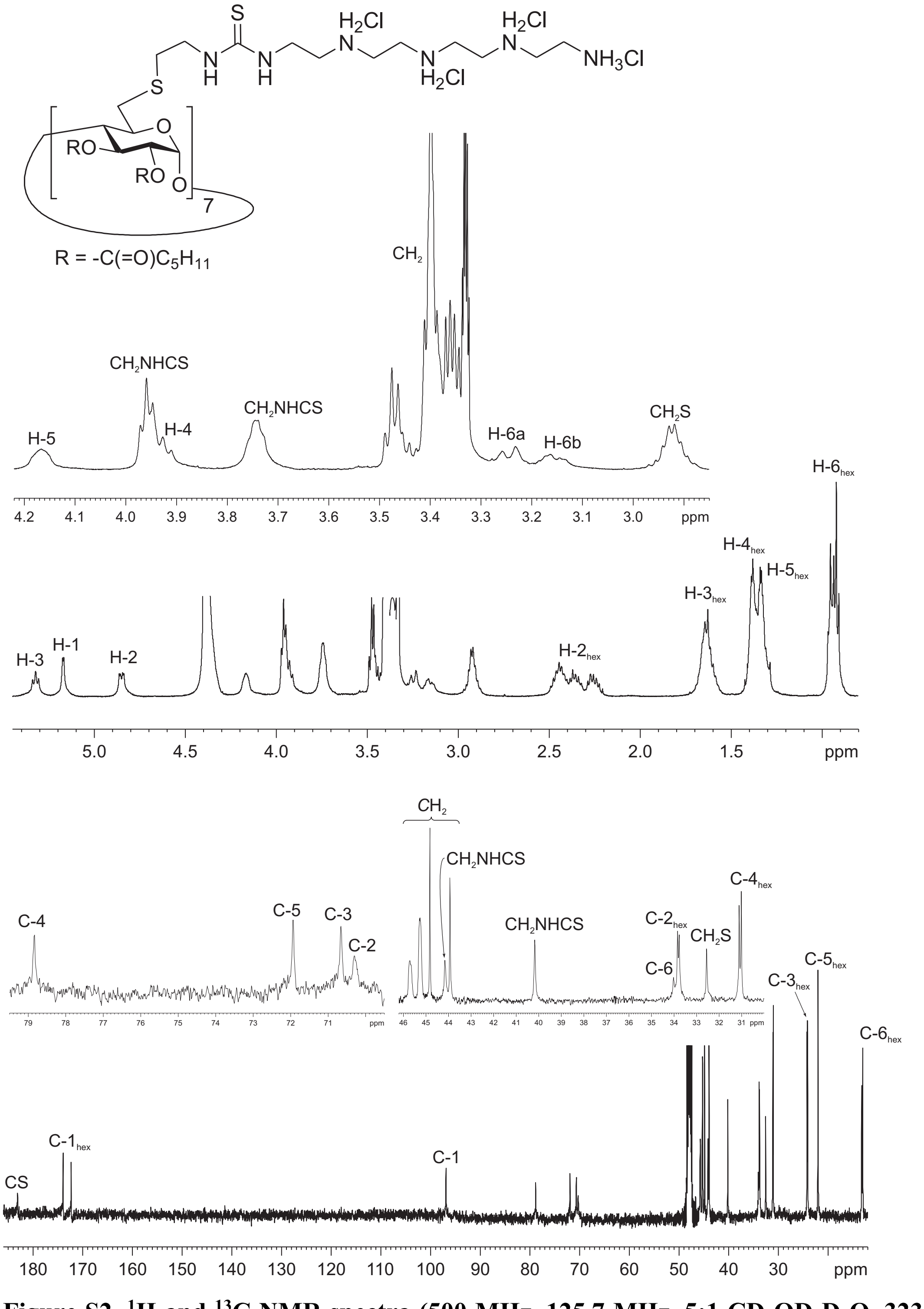

Figure S2. ^{1}H and ^{13}C NMR spectra (500 MHz, 125.7 MHz, 5:1 CD_3OD-D_2O, 323 K) of AMC11.

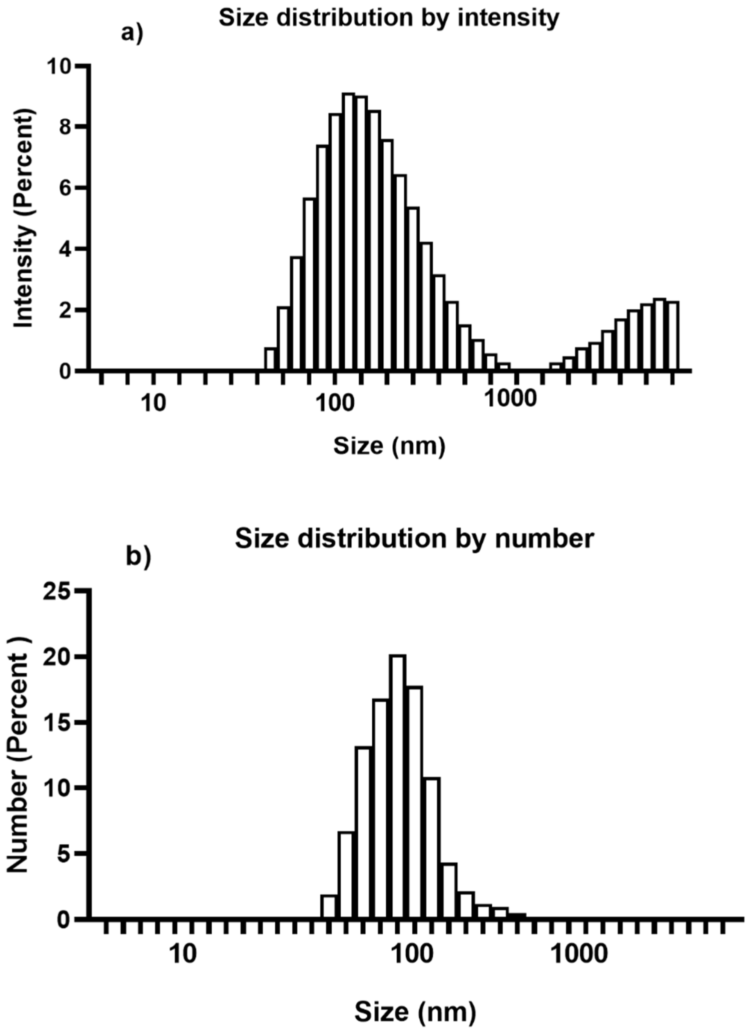

Figure S3 Size distribution by a) intensity and b) number of AMC11. Average size: 169.7 nm (Polydispersity Index, PI, 0.459). ζ-potential: 57.6 ± 1.39 (Mean ± S.E.M. of 4 measurements).

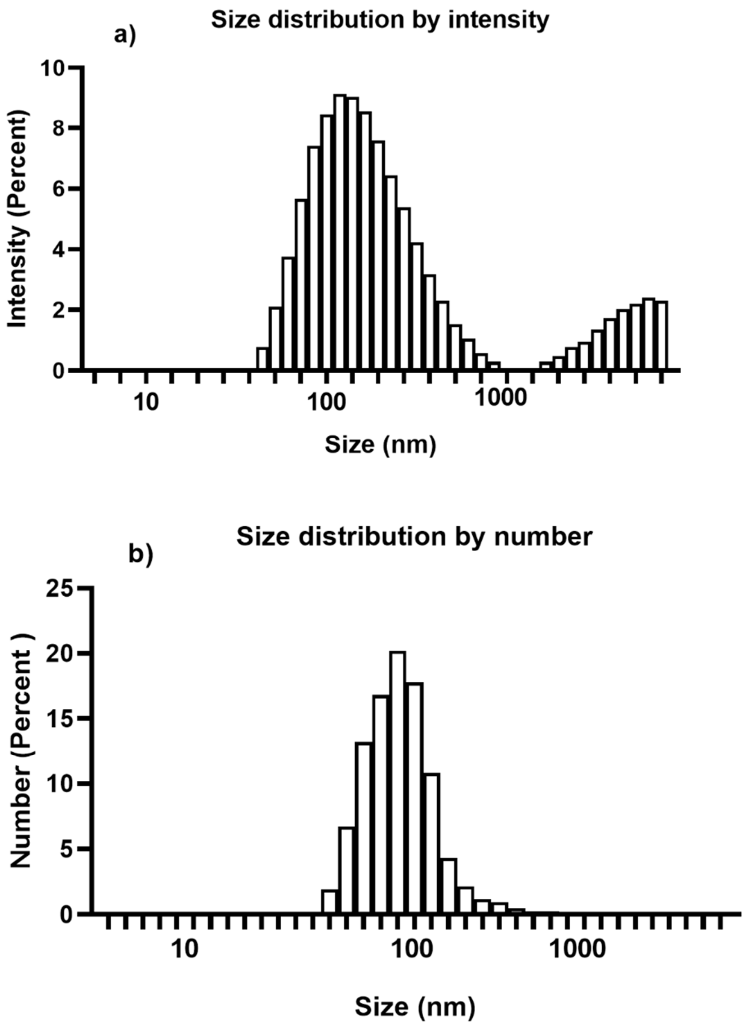

Figure S4. Size distribution by a) intensity and b) number of the nanoplex formed by AMC11 (1 µM) and SCR-siRNA (100 nM). Average size: 100.5 nm (PI 0.261). ζ-potential: 25.38 ± 0.46 (2.0 ± 0.03). Data represent a representative experiment (Mean ± S.E.M. of 3 measurements).

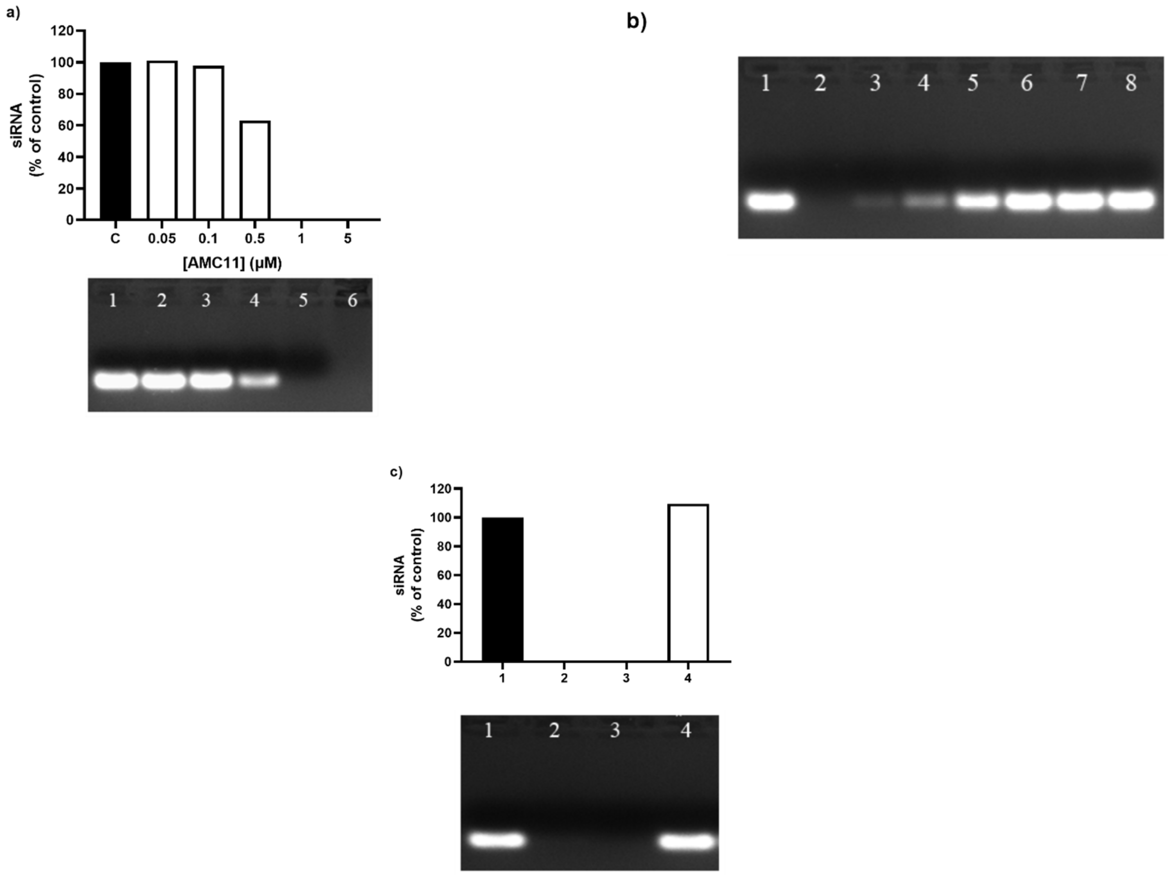

Figure S5. AMC11 interaction with siRNA. a) Gel retardation assay. A fix amount of SCR-siRNA (100 nM) was incubated with increasing concentrations of AMC11 ranging from 50 nM to 5 μM. The upper part of the graph represents the siRNA percentage found observed in each lane as compared to control value (C) in one experiment, repeated 3 times with similar results. b) Heparin displacement assay. Nanoplexes were formed at a N/P ratio of 6.66 (1 μM AMC11 and 100 nM siRNA) and exposed to increasing heparin concentrations ranging from 0.01 to 0.2 USP units (lanes 2 to 8). c) RNAse protection assay. Lane 1: free siRNA (control). Lane 2: SCR-siRNA 100 nM + AMC11 1 μM (nanoplex). Lane 3: free SCR-siRNA + RNAse + Heparin. Lane 4: Nanoplex + RNAse + Heparin. The upper graph represents SCR-siRNA percentage obtained in each lane compared with the control. The figure shows a representative experiment repeated 3 times with similar results.

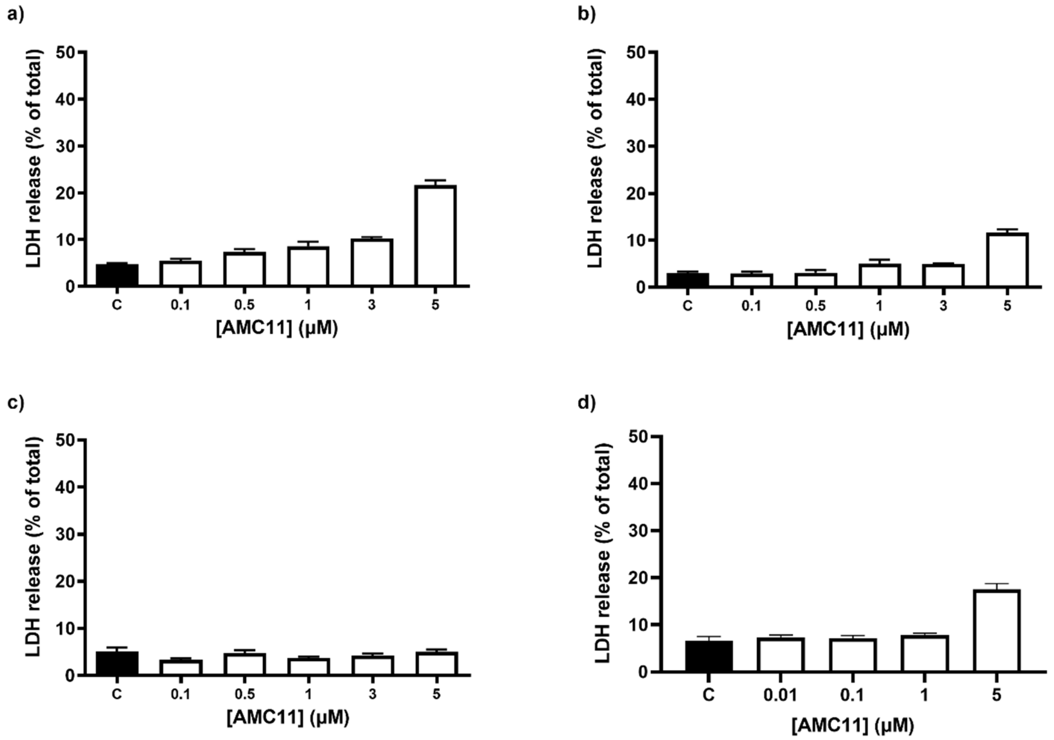

Figure S6. AMC11 toxicity on glioblastoma cell lines and astrocytes. Glioblastoma cell lines a) rat C6, b) mouse GL261, c) human T98G, and d) rat astrocytes were exposed to increasing AMC11 concentrations ranging 0.1 to 5 μM for 72 h. Then, the percentage of LDH release was determined as an index of cell death**.** Data represent mean ± S.E.M of 8 to 12 determinations.

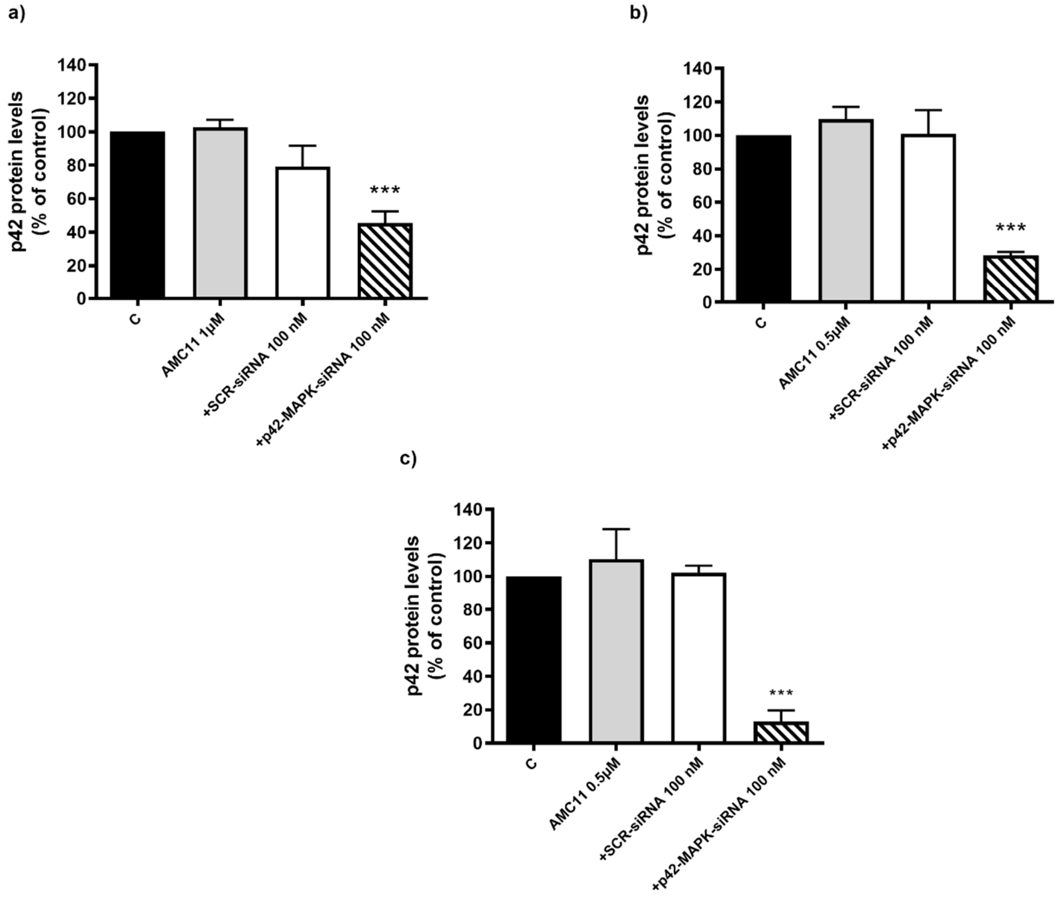

Figure S7. AMC11-siRNA knock down of p42-MAPK protein levels in a) C6, b) U87, and c) GL261 glioblastoma cell lines. Cells were exposed for 72 h to the indicated treatments and the specific protein levels determined as indicated in Material and Methods. Western blot bands show representative experiments for each experimental condition. Data represent mean ± S.E.M. of 4 experiments: ***p < 0.001 as compared to control values.

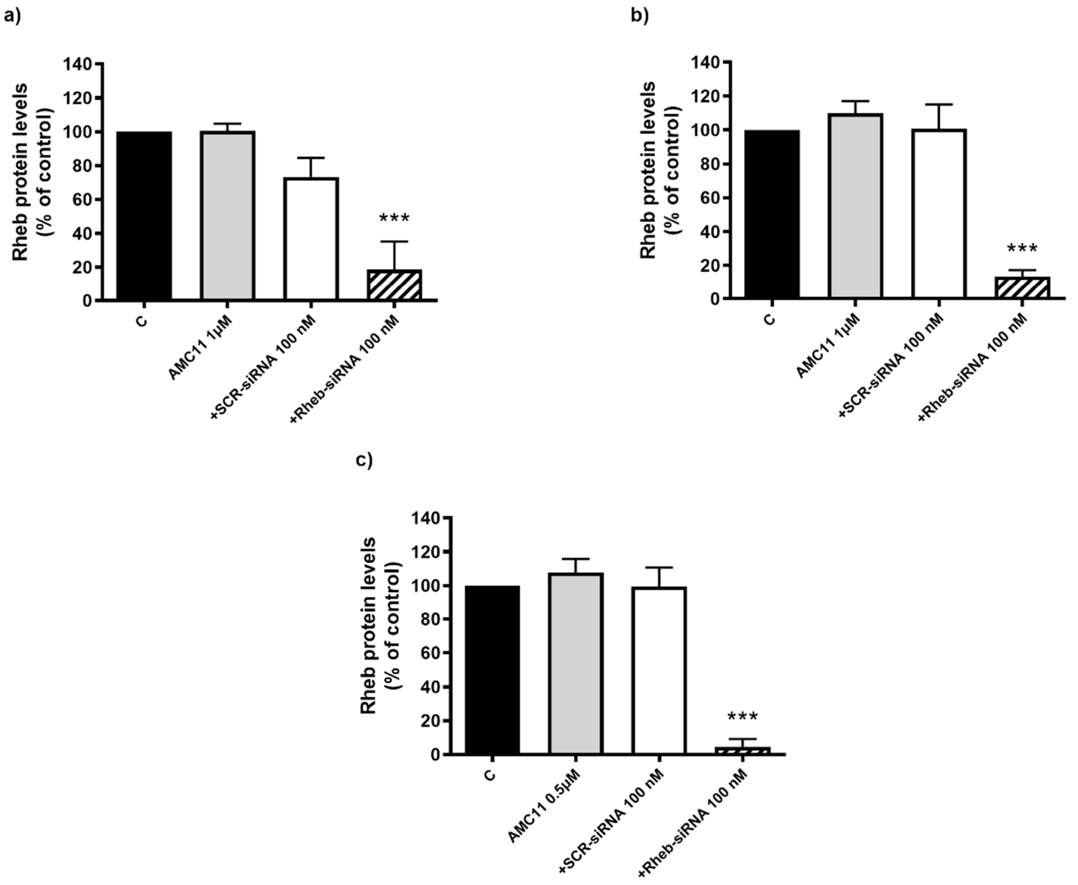

Figure S8. AMC11-siRNA knock down of Rheb protein levels in a) C6, b) U87, and c) GL261 glioblastoma cell lines. Cells were exposed for 72 h to the indicated treatments and the specific protein levels determined as indicated in Material and Methods. Western blot bands show representative experiments for each experimental condition. Data represent mean $\pm$ S.E.M. of 3-5 experiments: ***$p < 0.001$ as compared to control values.

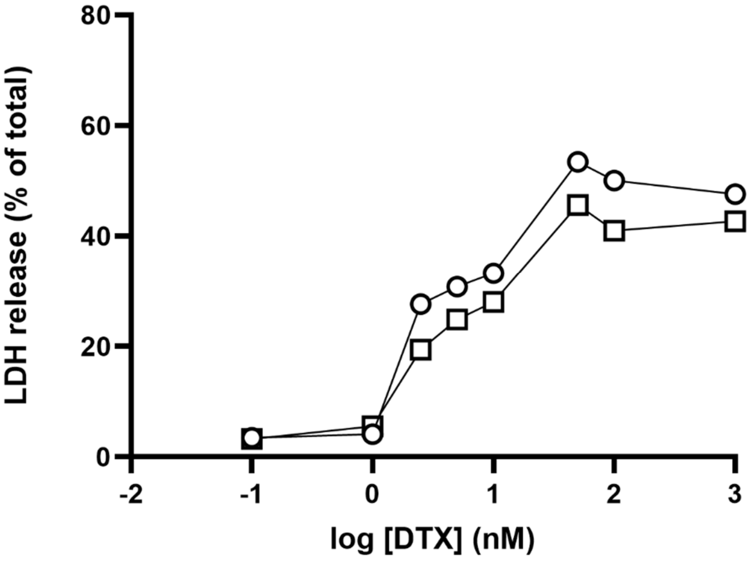

Figure S9. Dose-response curve of DTX-induced toxicity in in LNCaP (○) and PC3 (□) cells. Effect of increasing concentrations of DTX in LNCaP and PC3 after 72 h of incubation. Cells were exposed to increasing concentrations of DTX for 72 h and the percentage of LDH released to culture medium was determined. Data represent mean ± SEM of 28 to 377 experiments. Error bars are smaller than the symbol size.

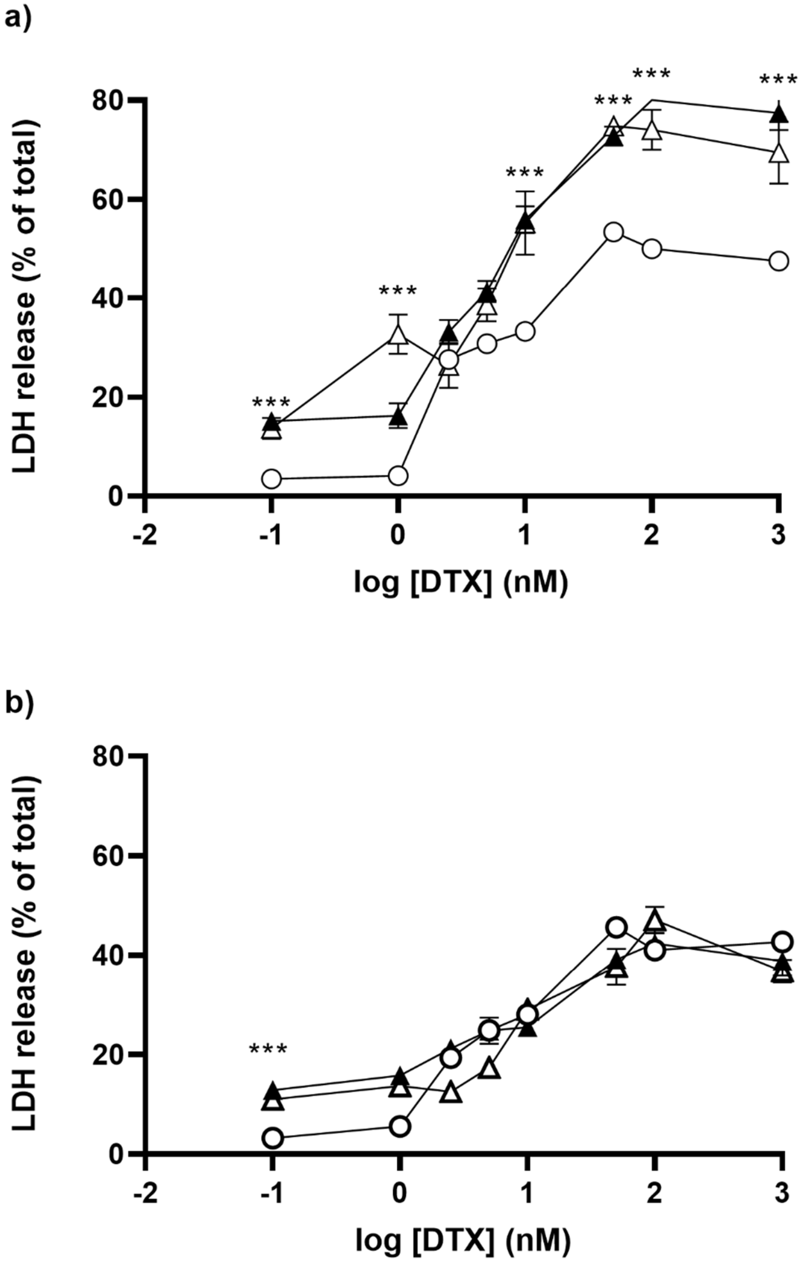

Figure S10. Effect of p42 (▲) or Rheb (Δ) knock down on DTX (○)-induced toxicity in a) LNCaP and b) PC3 cells. Data represent mean ± SEM of 8 – 22 experiments for siRNA treated cells. DTX dose-response curves in absence of siRNA are the same as shown in figure S9. If absent, error bars are smaller than the symbol size. ***$p < 0.001$ as compared to DTX in absence of siRNA.

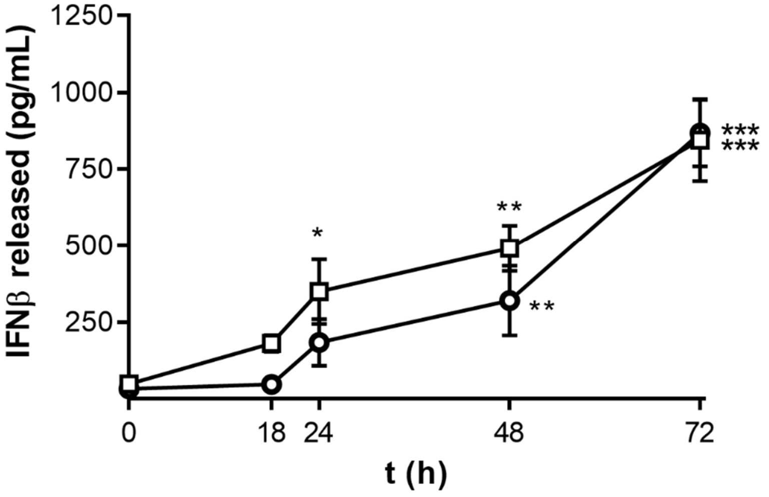

Figure S11. Time-course SCR-siRNA-induced production of IFNβ in LNCaP (○) and PC3 (□) cells. Cells were treated with nanoplexes formed by AMC11 (1 μM) + SCR-siRNA (100 nM) for the indicated times and IFNβ determined as indicated in Material and Methods. Data are expressed as mean ± S.E.M. from 6 to 12 determinations from at least three different experiments. *$p<0.05$; **$p<0.01$; ***$p<0.001$ compared with untreated cells.

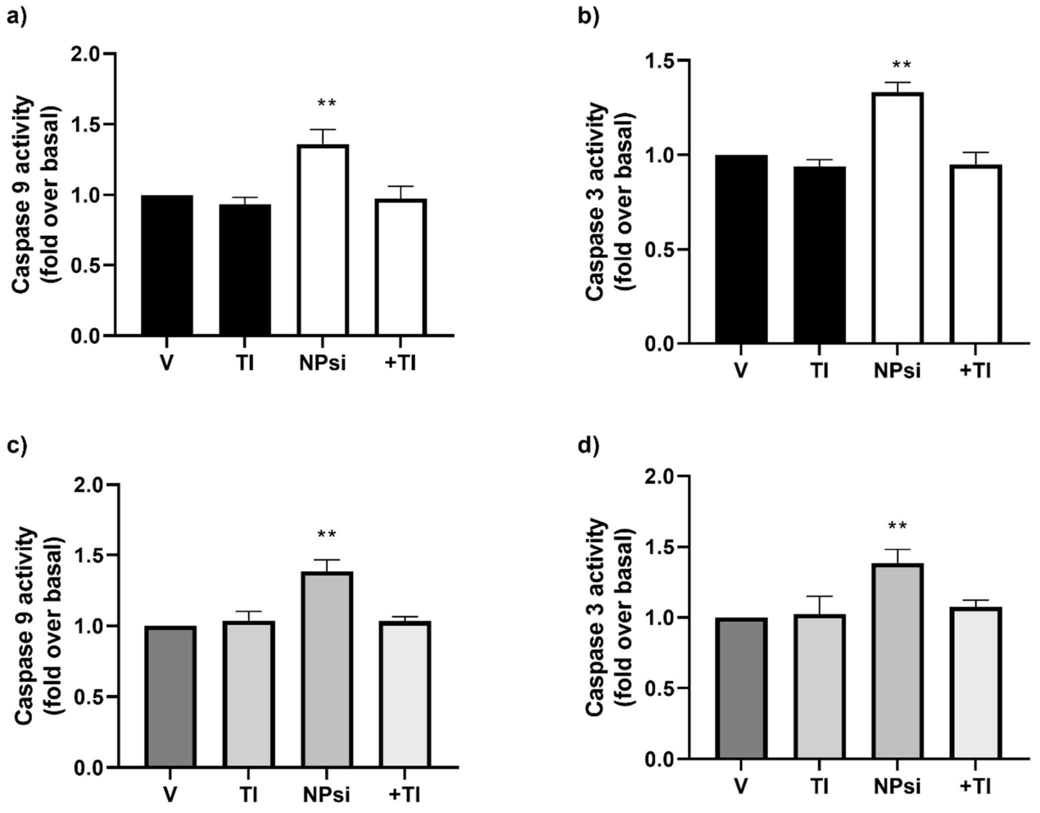

Figure S12. Scramble-siRNA-induced activation of caspases in LNCaP (a, b) and PC3 (c, d). cell lines. Cells were treated with nanoplexes formed by AMC11 (1 µM) and SCR-siRNA (NPsi, 100 nM) for 24 h and Caspase 3 (a, c) and 9 (b, d) enzymatic activities were determined in presence and absence of a TLR3 inhibitor (TI, 10 µM). Data are expressed as mean ± S.E.M. from 4 to 6 experiments. **p<0.01 compared with control-treated cells.

Original western blot gels

Figure 5

p42-MAPK

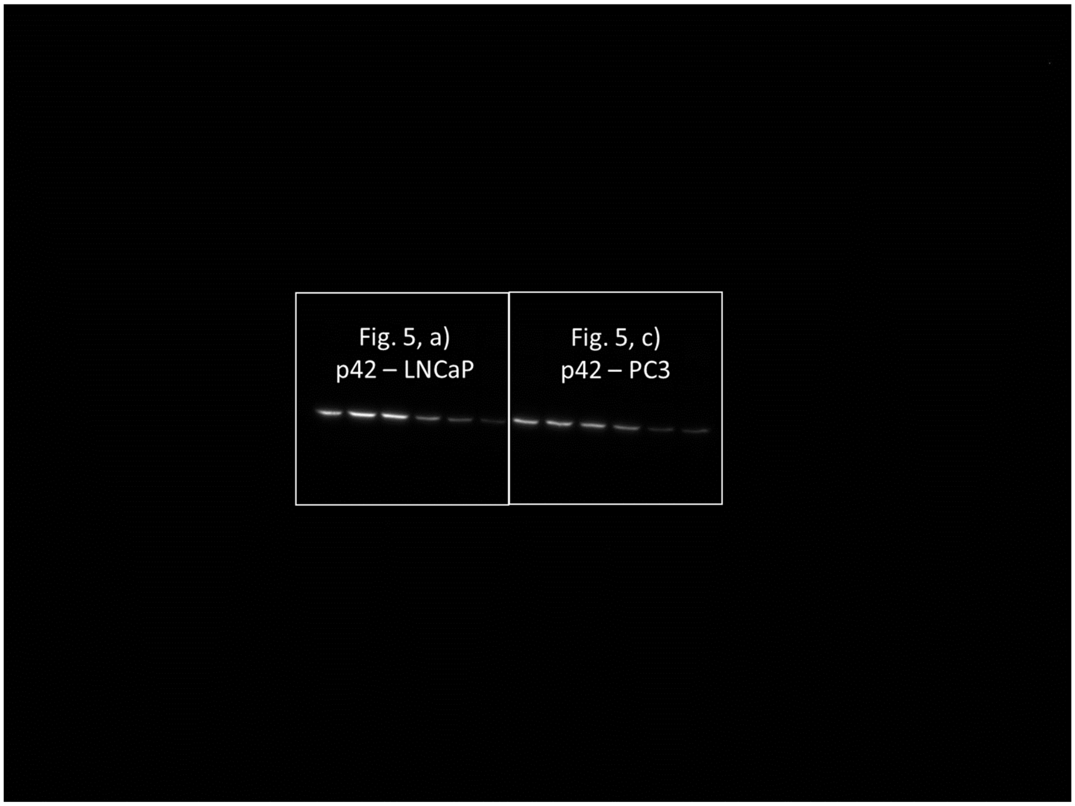

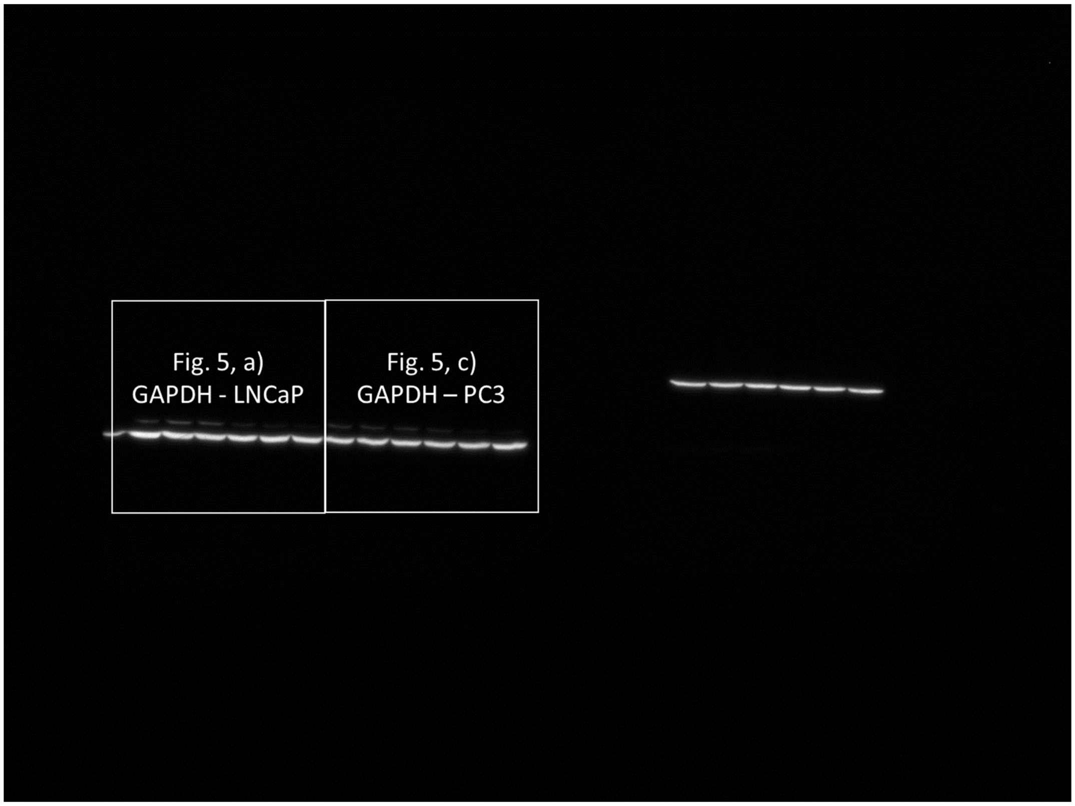
Fig. 5, a)
GAPDH - LNCaP
Fig. 5, c)
GAPDH – PC3

Rheb

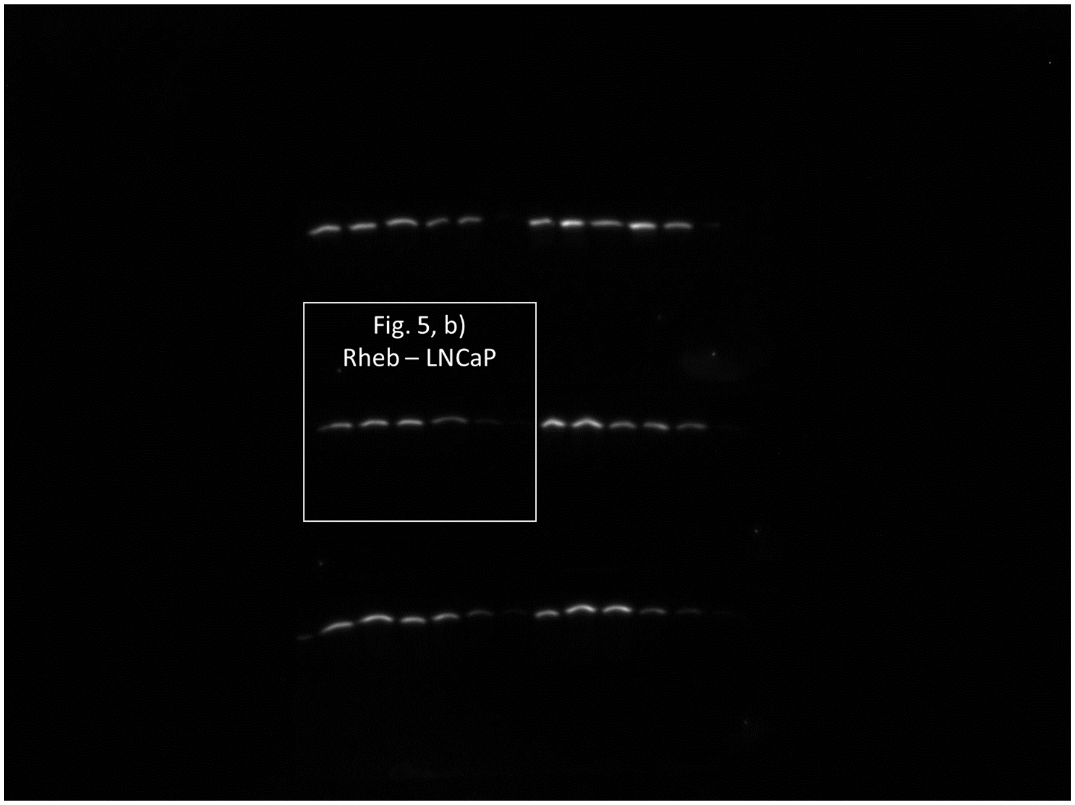

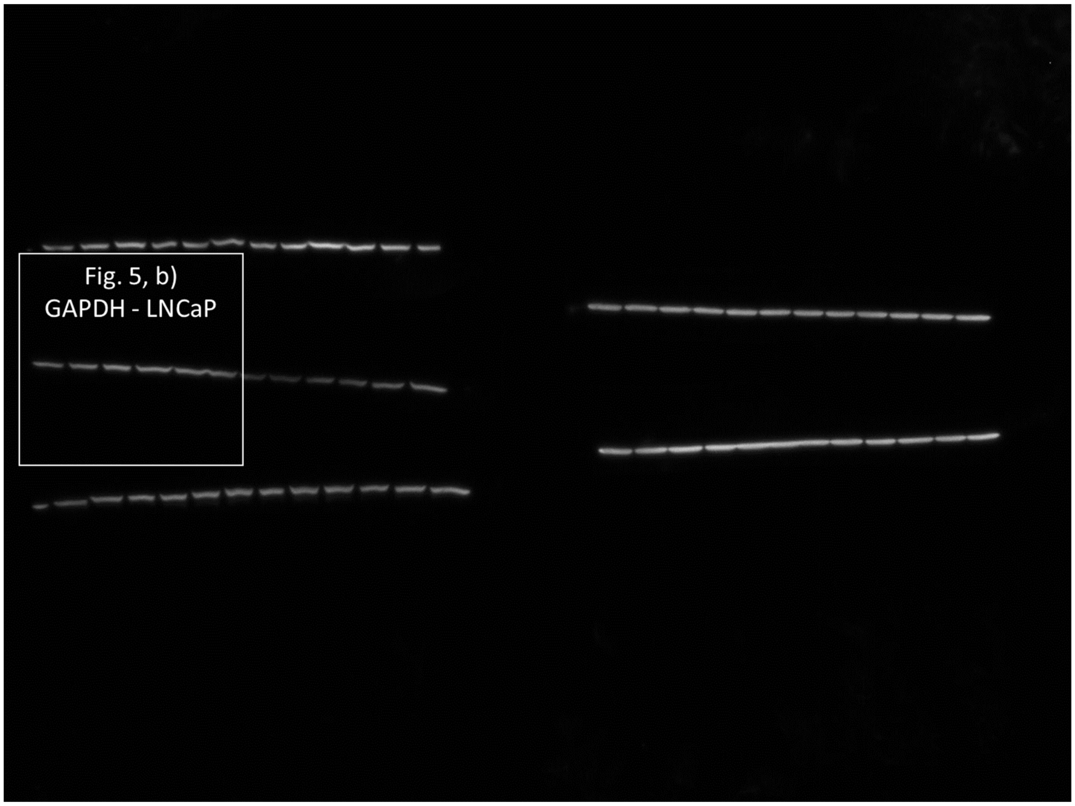

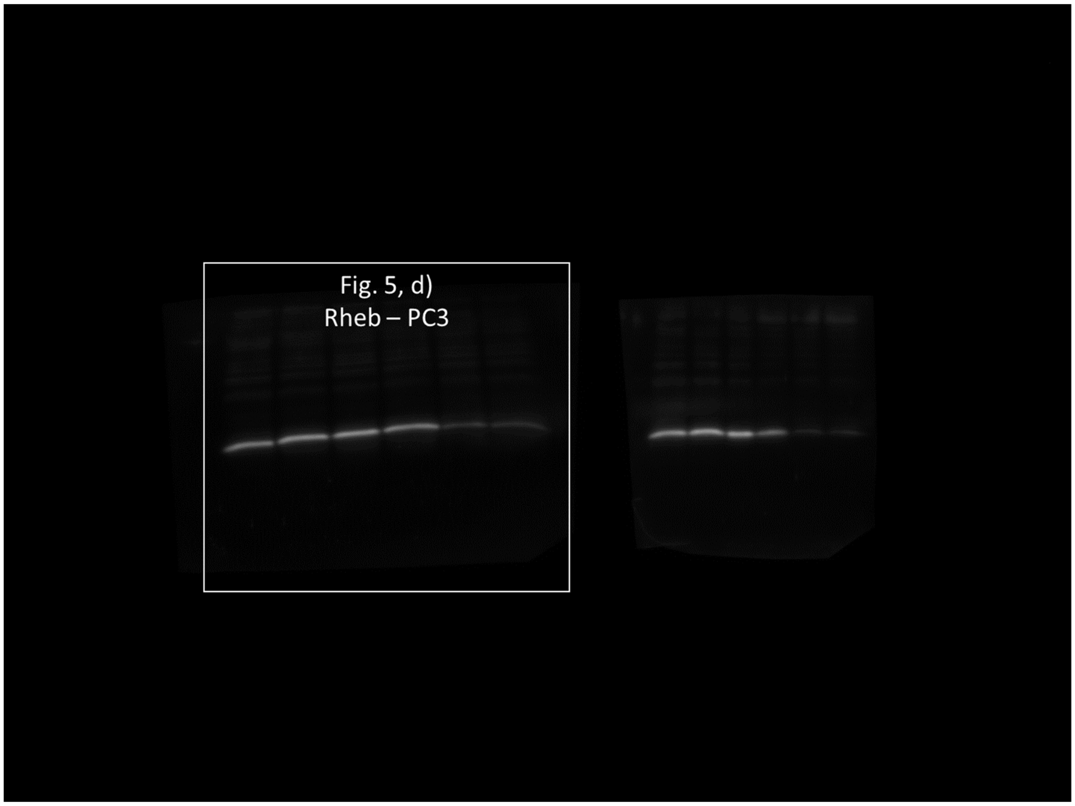
Fig. 5, d)
Rheb – PC3

Figure 7

Figure 7a. pIRF3

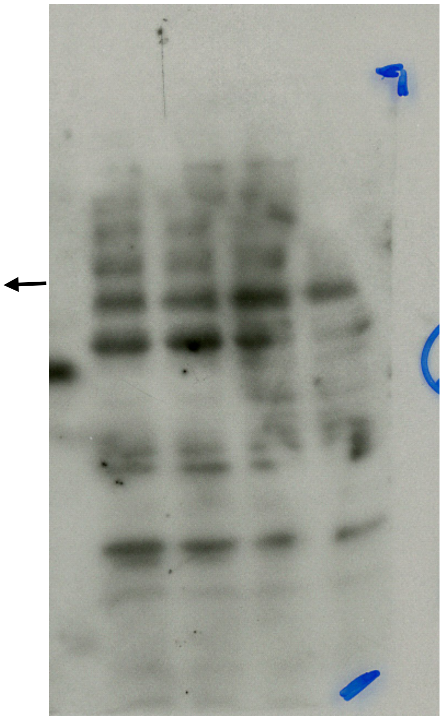

Figure 7a. IRF3

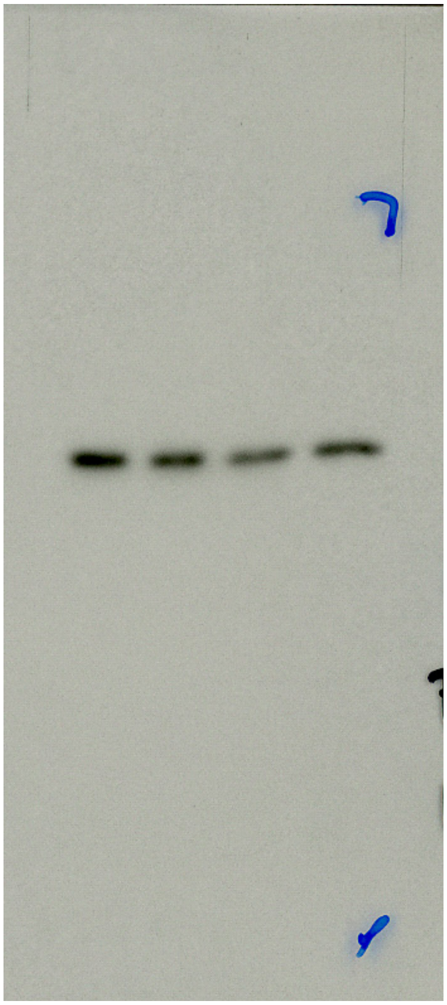

Figure 7a. Tubulin

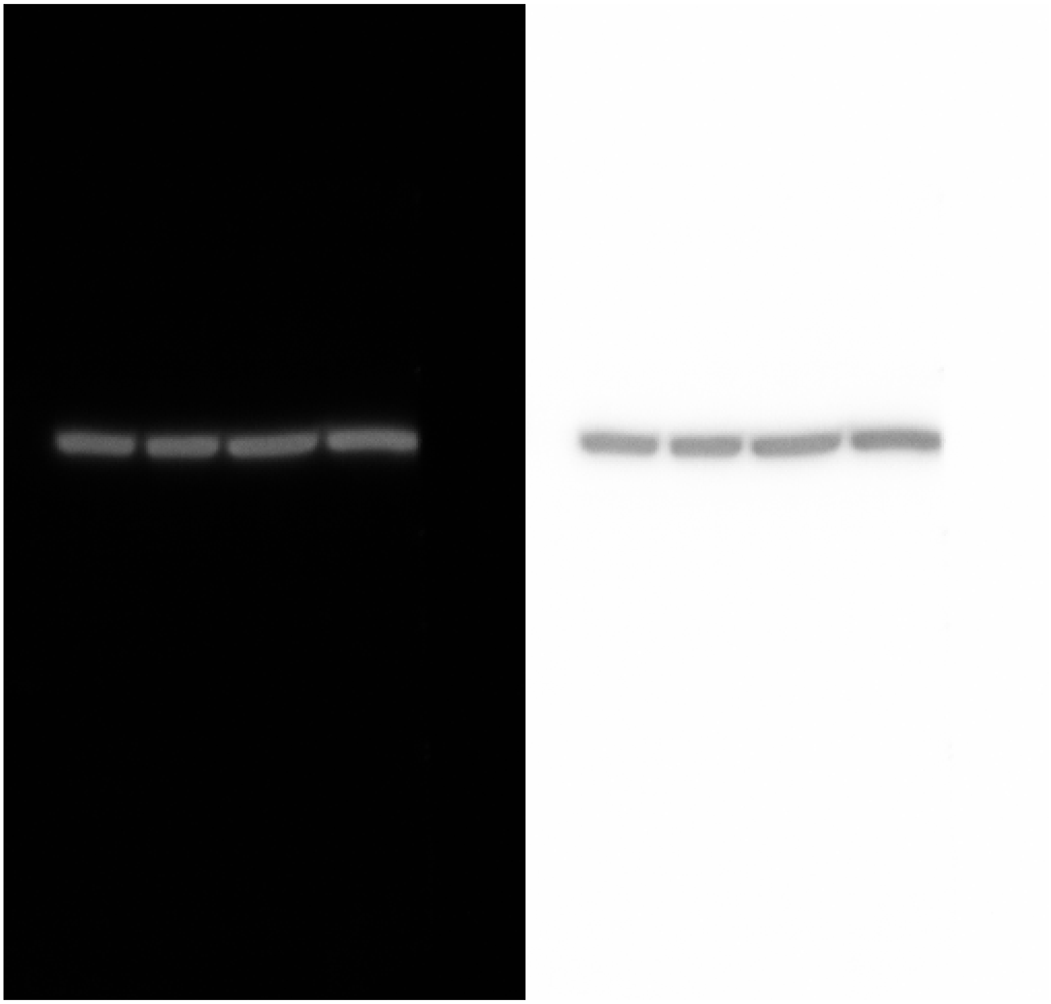

Figure 7b. pIRF3

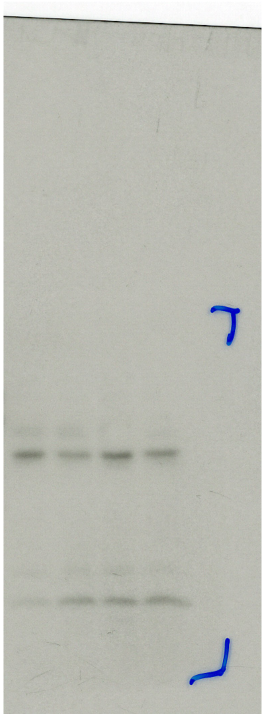

Figure 7b. IRF3

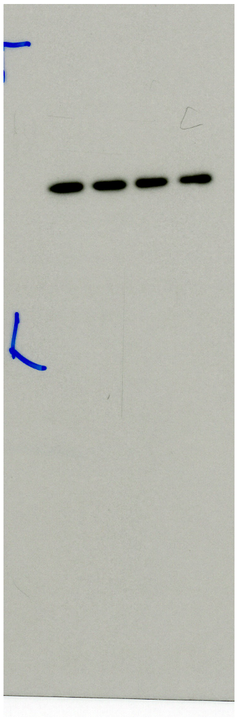

Figure 7b. H2A

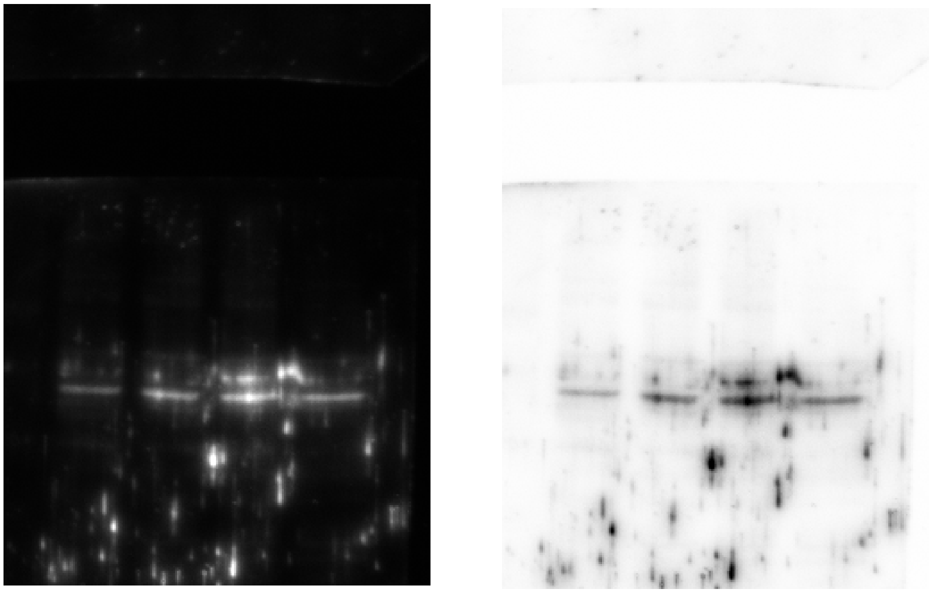

Figure 7c. pIRF3

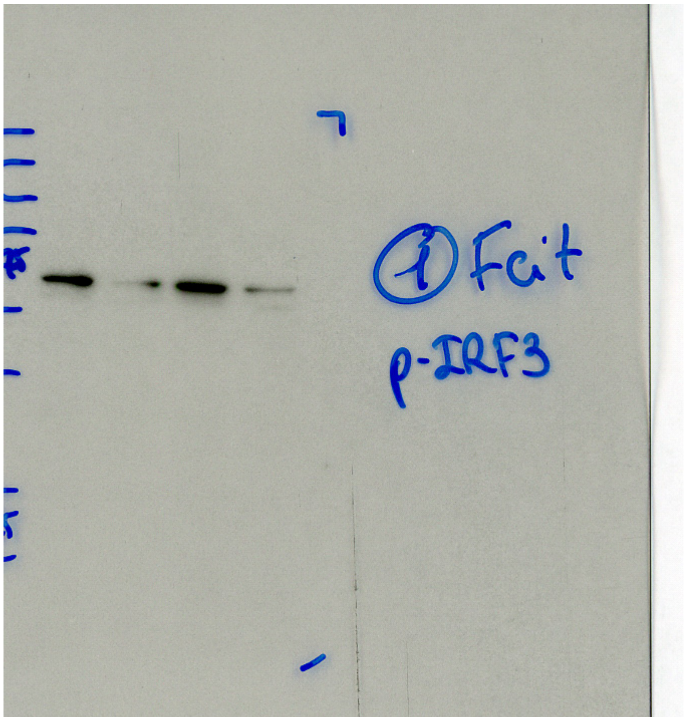

Figure 7c. IRF3

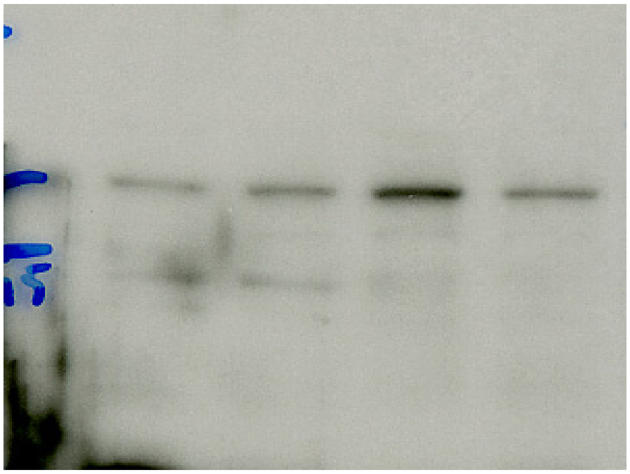

Figure 7c. Tubulin

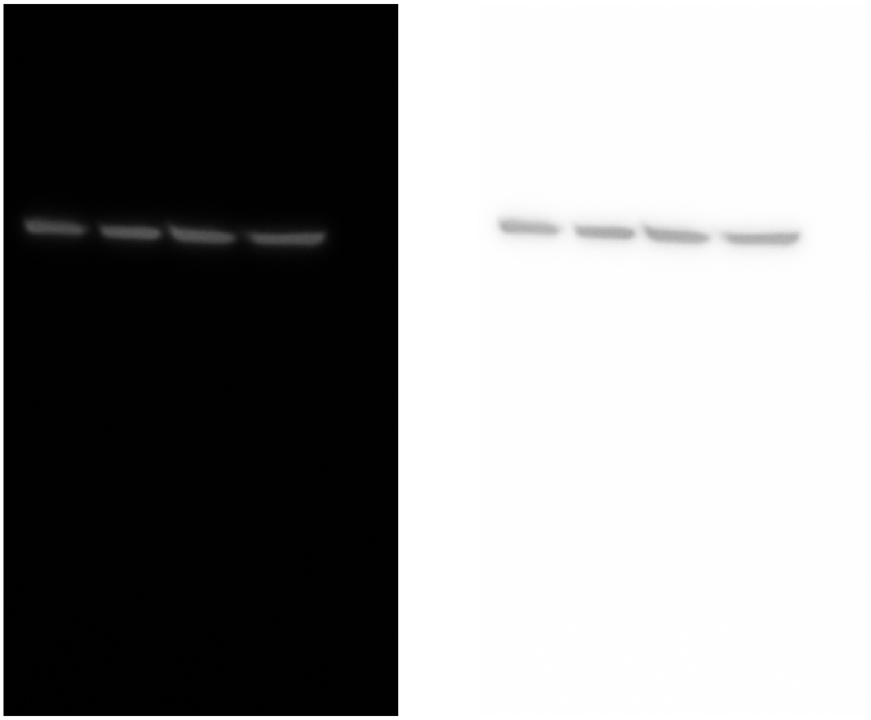

Figure 7d. pIRF3

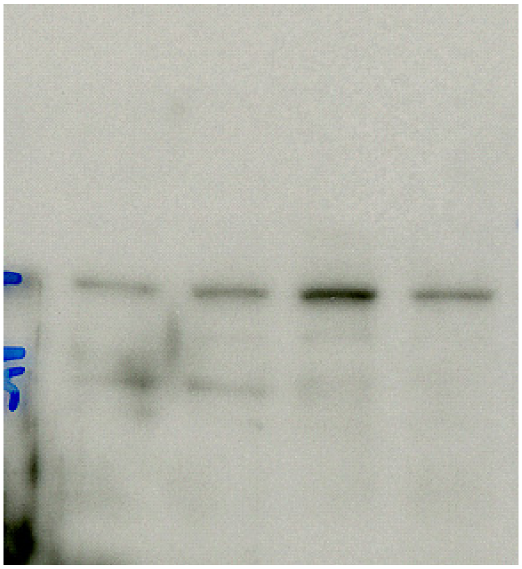

Figure 7d. IRF3

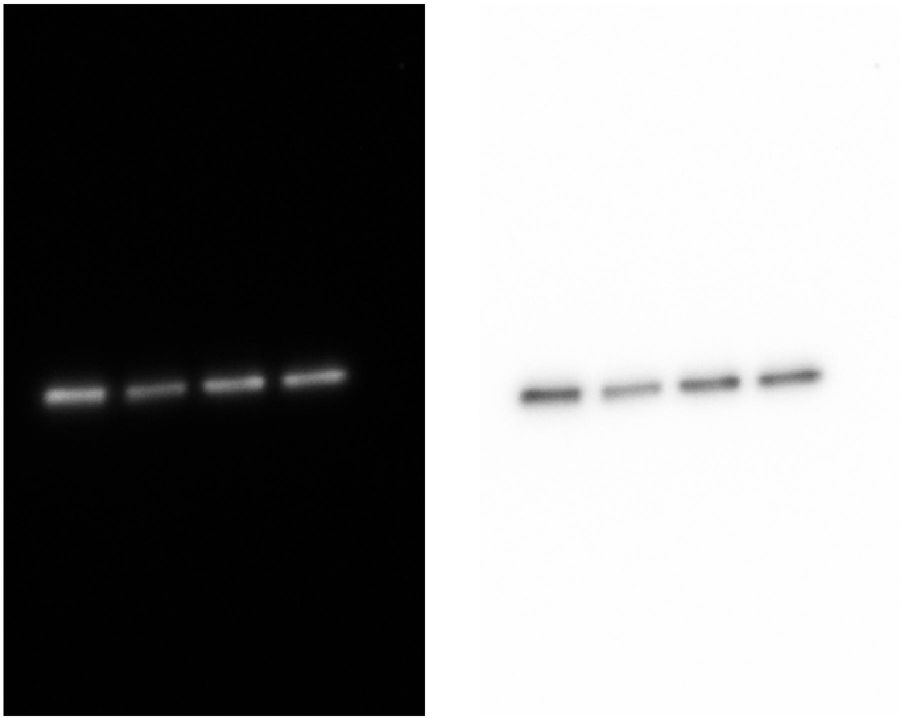

Figure 7d. H2A

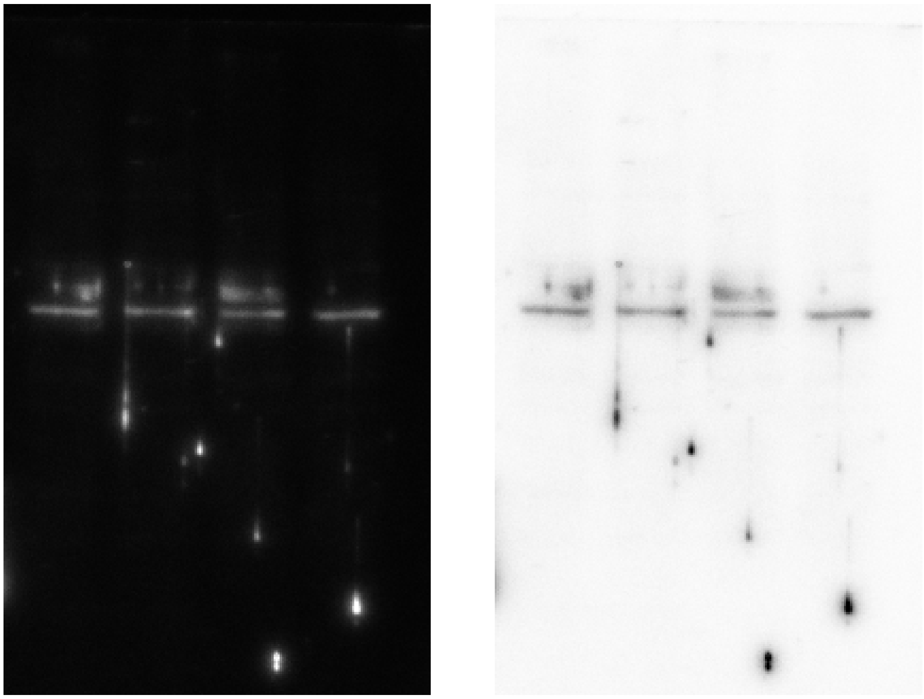

Supplement: Supplementary file 1 [file pharmaceutics-14-02424-s001.zip › pharmaceutics-2011750-supplementary.pdf]
